# Multiplexed Nanophotonic Sensor Arrays for Time-resolved Biomolecular Analysis

**DOI:** 10.1016/j.bios.2026.118669

**Published:** 2026-04-07

**Authors:** Lisa M. Miller, Christopher P. Reardon, Kathryn G. Leslie, Callum D. Silver, Joshua S. Male, Clare S. Mahon, Thomas F. Krauss, Steven Johnson

**Affiliations:** 1 School of Physics and Technology, University of York, Heslington, York, YO10 5DD, UK; 2 Phorest Diagnostics Ltd., School of Physics and Technology, University of York, Heslington, York, YO10 5DD, UK; 3 Department of Chemistry, Durham University, Durham DH1 3LE, UK

**Keywords:** Chirped guided-mode resonance, nanophotonic sensor arrays, label-free biosensing, binding kinetics, CMOS-compatible fabrication, multiplexed detection, point-of-care diagnostics

## Abstract

Complex diseases arise from networks of interacting biomolecules, yet most analytical technologies measure only a limited number of interactions simultaneously. Here, we present a chirped guided-mode resonance (cGMR) photonic biosensor array for multiplexed, label-free analysis of biomolecular kinetics across hundreds of sensing sites. The platform integrates 322 photonic sensors on a single chip that are fabricated using a CMOS process ensuring high reproducibility (wavelength sensitivity of 36.8 pixels/nm with a standard deviation of 1.37 pixels/nm). Shifts in the photonic resonance due to molecular binding are recorded using a conventional CMOS camera allowing time-resolved and simultaneous measurements across the entire array. We demonstrate the platform’s versatility by monitoring real-time binding of antibodies, aptamers, and synthetic glycopolymers where the parallel measurements reveal distributions of binding responses arising from surface heterogeneity and multivalency that are inaccessible to conventional, single biosensor platforms. As a model, wheat germ agglutinin binding to a GlcNAc-displaying glycopolymer yielded sub-micromolar equilibrium dissociation constants (*K*_D_ = 28-564 nM). Crucially, the array successfully resolves the kinetics of lectin-glycopolymer binding interactions using a portable optical setup, even in a complex biological matrix. By combining scalable CMOS fabrication with portable, highly parallel interrogation, this cGMR array provides a robust route toward high-throughput biomolecular profiling in point-of-care diagnostics and drug discovery.

## Graphical abstract

**Figure:**
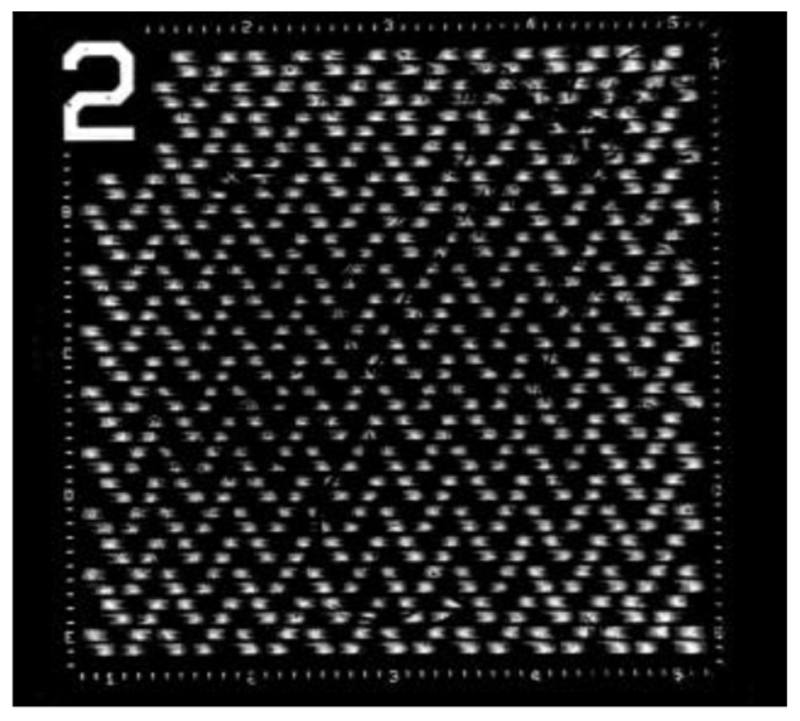

## 1 Introduction

Label-free biosensors that quantify molecular interactions are central to applications in clinical diagnostics, precision medicine, environmental monitoring and drug discovery. As disease states often arise from complex and heterogeneous molecular processes, diagnostic insight increasingly depends on measuring panels of biomarkers rather than single analytes. Technologies capable of multiplexed detection are essential for capturing this complexity and for enabling more accurate, personalised decision-making. Surface-affinity biosensors typically combine an immobilised recognition interface – formed from antibodies, aptamers or other selective binders - with a transducer, commonly electrical or optical, that converts binding into a measurable signal. Among these, photonic biosensors offer high sensitivity, rapid response and intrinsic compatibility with complementary metal-oxide-semiconductor (CMOS) manufacturing methods, enabling the fabrication of highly reproducible devices manufactured at the wafer scale (Azadmousavi and Ghafar-Zadeh, 2024; Krauss *et al*. 2024). This compatibility supports low-cost production, device uniformity and long-term reliability, while also allowing dense arrays of sensors to be integrated on a single chip for parallel biomolecular detection.

A range of photonic sensor arrays have been developed to address the need for multiplexed measurements, including platforms based on silicon photonic microring resonators, photonic-wire structures and photonic crystal devices (Densmore *et al*. 2009; De Vos *et al*. 2009; Iqbal *et al*. 2010). For example, commercial systems such as the Genalyte microring resonator platform highlight the translational potential of this approach, performing label-free immunoassays across 64-128 microring sensors (Mudumba *et al*. 2017). However, these and other arrays rely on coupling light into and out of individual sensing elements, imposing stringent requirements on optical alignment and stability preventing deployment in low-cost or portable formats. Light coupling combined with the need for spectral analysis also imposes limits on parallel readout and on the measurement temporal resolution. As a result, current photonic arrays struggle to capture the kinetics of biomolecular interactions, such as association and dissociation rates, which are critical for characterising drug–target interactions and probing low-affinity, transient protein interactions central to signalling and pathogen–host recognition (Wright *et al*. 2010).

Here we present a chip-scale photonic sensor array designed to overcome these limitations in high-throughput, real-time, label-free biosensing. The platform integrates 644 chirped guided-mode resonance (cGMR) gratings, operating as 322 differential, mechanical-noise-tolerant sensor pairs, all interrogated in parallel with high temporal resolution. Guided-mode resonance sensors exploit optical resonances in wavelength-scale gratings to achieve sensitive refractive-index detection. In the cGMR architecture, the spectral information is spatially encoded, enabling plane-wave excitation using a standard LED and readout with a conventional CMOS camera, thereby eliminating fibre coupling, spectrometers and scanning optics (Triggs *et al*. 2017). The simplicity and robustness of the coupling design enable a compact, mechanically-robust and low-cost instrument architecture suitable for portable operation (Gilroy *et al*. 2025; Drayton *et al*. 2020). We demonstrate the performance of the cGMR array and its compatibility with antibodies, aptamers and synthetic glycopolymers, enabling real-time measurement of complex recognition processes such as carbohydrate–lectin interactions. By combining nanoscale optical resonances with wafer-scale fabrication and massively parallel interrogation, this platform establishes a new class of reliable, high-density photonic biosensor arrays for multiplexed molecular analysis, with implications for personalised diagnostics, bioreceptor discovery and drug screening.

## 2 Results and discussion

### 2.1 Nanophotonic Array Design and Characterisation

**Figure 1:**
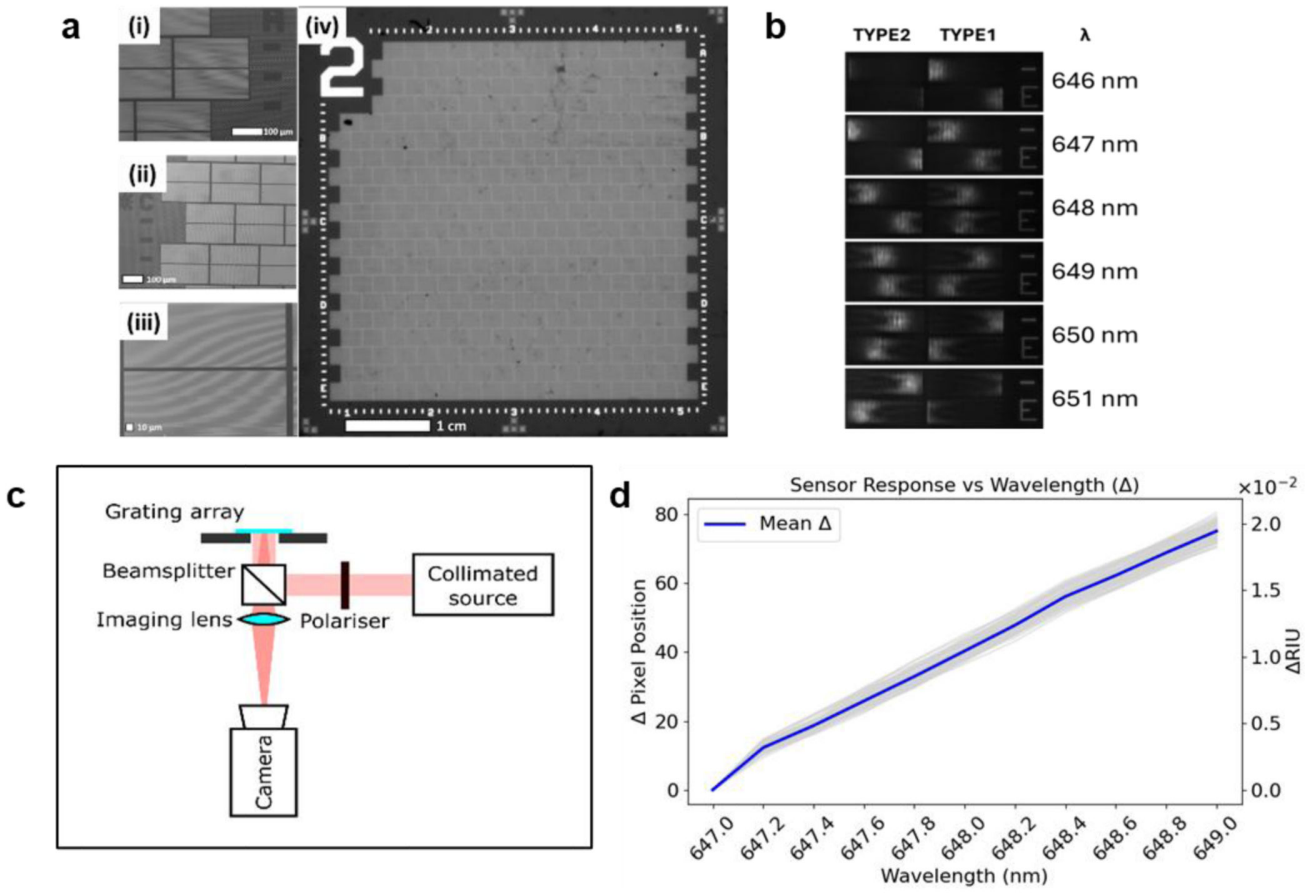
Photonic Array Design and Characterisation: (a) SEM micrographs of the array, detailing: (i) and (ii) tiled pattern of sensor pairs; (iii) an individual pair of cGMRs; and (iv) bright field image of the full sensor array chip. (b) Resonance bar movement with changing wavelength (from 646 - 651 nm, top to bottom) for type 1 and type 2 sensors. (c) Schematic of the optical setup for measuring cGMR sensors. (d) Array response recorded as resonance shift (in camera pixels, per grating) against wavelength (n= 79) showing mean shift (blue) and individual sensors (light grey); secondary y-axis showing equivalent change in refractive index units (ΔRIU).

The array was designed to feature 322 individual sensors in which each sensor consists of two cGMRs, which function as a pair. The pair allows for internal referencing, removing noise from mechanical factors such as vibration (Li *et al*. 2024). The array is fabricated on a 5.4 × 5.4 cm chip in a CMOS foundry, with each cGMR being only 250 × 100 μm in size (Fig. 1a). A key design trade-off in the miniaturisation of the cGMR gratings is the balance between dynamic range and refractive index (RI) sensitivity; reducing the footprint narrows the resonance bandwidth, limiting dynamic range unless sensitivity is sacrificed. Our approach resolves this trade-off by combining two sensor designs with overlapping resonance ranges. This architecture preserves the high RI sensitivity characteristic of larger cGMR sensors (Figs. S1b, S2) while extending the dynamic range beyond the limits of a single-element device. In detail, the photonic array incorporates two distinct cGMR sensor designs, each engineered with a different resonance window by tuning the grating period (type 1: 420-424 nm; type 2: 421-425 nm). The inclusion of two overlapping resonance ranges increases the effective operational bandwidth of the array while simultaneously increasing the tolerance for operating with a fixed-wavelength light source. Both type 1 and type 2 cGMRs provide a usable spectral window of approximately 4 nm, while the 3 nm overlap between them yields a total functional wavelength range of 5 nm (Fig. 1b). The associated cGMR optical setup requires only basic optical components: a collimated light source, polariser, beamsplitter, imaging lens, and camera (Fig. 1c). The platform is thus inexpensive, straightforward to implement and robust in a variety of experimental conditions and allows the response of each cGMR element in the array to be recorded in parallel.

Characterisation of the array was initially performed using a laboratory optical instrument, with 79 sensor pairs within the field of view (158 individual cGMRs). The wavelength sensitivity of each sensor pair was assessed by measuring the shift in the position of the optical resonance (Δpixel position) as the illumination wavelength was scanned between 647 nm to 649 nm in 0.2 nm increments, corresponding to an effective refractive index dynamic range of approximately 2 × 10^−2^ refractive index units (RIU) (Fig. 1d). We note a highly consistent response, with wavelength sensitivities averaging 36.8 pixels/nm with a standard deviation of only 1.37 pixels/nm, demonstrating minimal inter-sensor variability across the array associated with the high reproducibility provided by the CMOS manufacturing. Unfunctionalised sensor measurements in water confirm high baseline stability and low intrinsic variability (Fig. S1). Based on the mean array noise (3σ, Fig. S1e), the limit of detection (LOD) and quantification (LOQ) were determined as 4.93 × 10^-4^ RIU and 1.64 × 10^-3^ RIU, respectively.

### 2.2 Measuring and Quantifying Binding Interactions

**Figure 2:**
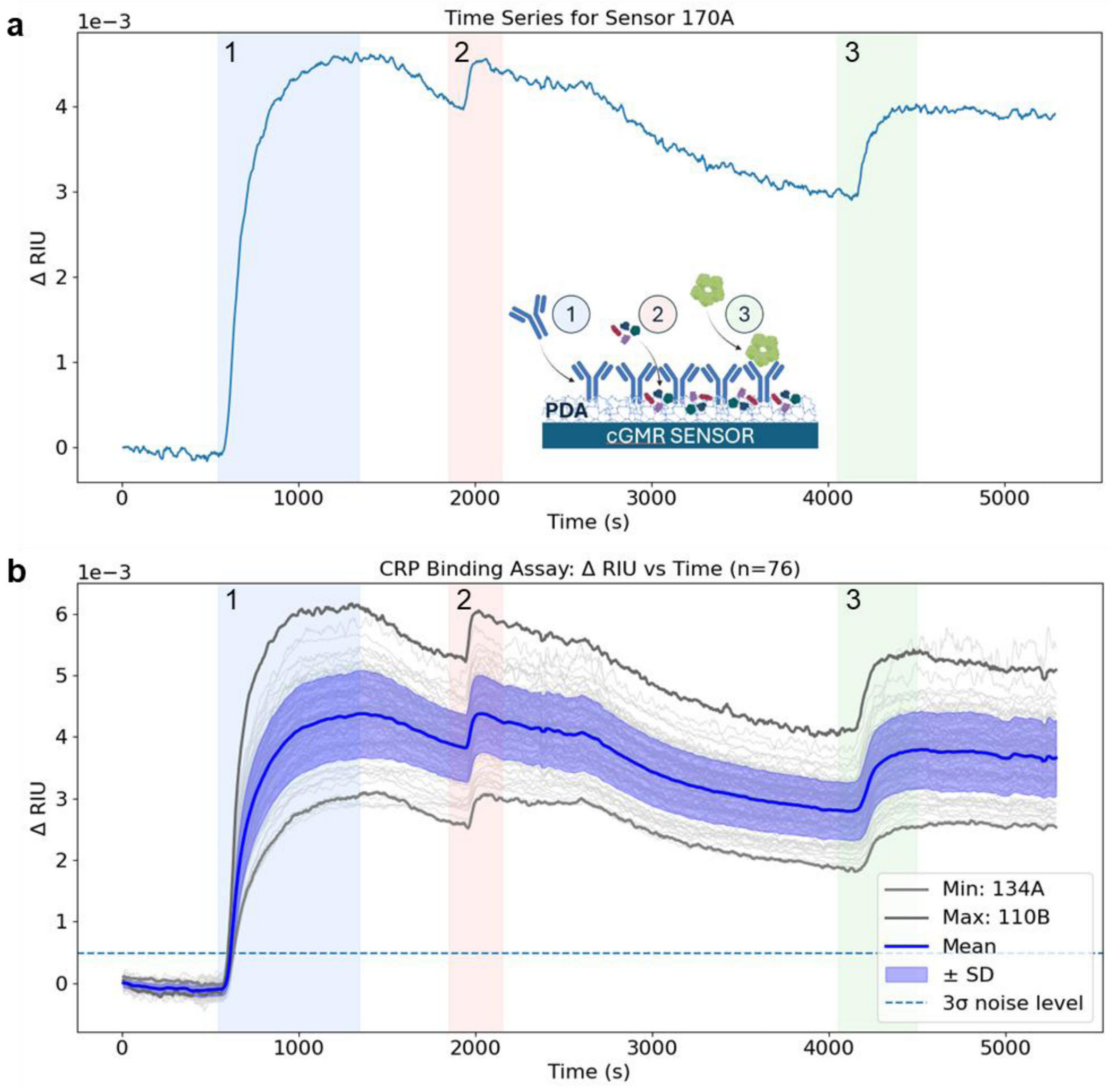
CRP binding assay (n = 76): (a) Single cGMR sensor (170A) resonance during functionalisation and CRP binding, with inset schematic showing anti-CRP immobilisation onto the PDA functionalised sensor (step 1), blocking of exposed surface sites (step 2), and finally CRP binding (step 3). (b) Experimental CRP binding responses of 76 cGMR sensor pairs (light grey), the mean ± SD (blue), and the minimum and maximum response (dark grey).

To characterise binding interactions across the array, we measured the binding of an antigen to its corresponding antibody immobilised on the sensor surface, using the inflammatory biomarker C-reactive protein (CRP) as an exemplar. We used a polydopamine (PDA) adhesion layer, which supports attachment of biomolecules through a combination of covalent and non-covalent interactions (Lee *et al*. 2007; Bakshi *et al*. 2024). Following antibody immobilisation (Fig. 2a, step 1), the remaining surface sites were passivated using SuperBlock™ (Fig. 2a, step 2). Antigen binding was then measured by flowing a 20 μg mL^−1^ CRP solution across the array (Fig. 2a, step 3). All steps were performed under continuous flow (100 μL min^−1^) using a bespoke microfluidic cartridge (Fig. S19).

All sensors exhibited clear resonance shifts during each binding step, enabling surface density calculations (SI S1.2–1.3). While individual traces capture the general binding sequence (Fig. 2a), array-level data reveals significant sensor-to-sensor variability in functionalisation and binding (Fig. 2b). This variance far exceeds instrumental noise and measured sensor-to-sensor variability (Fig. 1d) and is likely associated with differences in molecular surface coverage and molecular orientation. This observed and quantifiable heterogeneity highlights an additional value of our multiplexed platform; the ability to provide statistical confidence in the analytical response which is inherently limited in singleplex biosensor platforms.

Beyond equilibrium measurements, our platform captures time-resolved binding dynamics inaccessible to end-point assays. A single-exponential function (SI S1.4) was fit to the temporal evolution of the sensor signal to determine the apparent rate constants (*k*_app_) for each functionalisation and binding step. Antibody immobilisation (Step 1) showed the largest signal change and rapid kinetics (ΔRIU = 4.16 × 10^−3^, *k*_app_ = 0.0374 s^−1^), indicating efficient attachment to the PDA surface. Blocking (Step 2) yielded a smaller response with faster initial saturation (ΔRIU = 5.62 × 10^−4^, *k*_app_ = 0.2732 s^−1^), while subsequent CRP antigen binding (Step 3) exhibited intermediate dynamics (ΔRIU = 9.76 × 10^−4^, *k*_app_ = 0.0649 s^−1^) consistent with specific molecular recognition.

To demonstrate platform versatility, we also evaluated three distinct biorecognition chemistries: antibodies, aptamers, and synthetic glycopolymers. These represent complementary tools for detecting diverse targets, from proteins and small-molecule drugs to carbohydrate-mediated pathogen binding (Leslie *et al*. 2024a, 2024b; Johnson *et al*. 2026). Binding experiments showed clear resonance shifts on functionalised sensors with no measurable response on parallel controls, confirming high specificity (see SI Section S5 for protocols and data).

### 2.3 Label-Free Molecular Interaction Mapping

**Figure 3:**
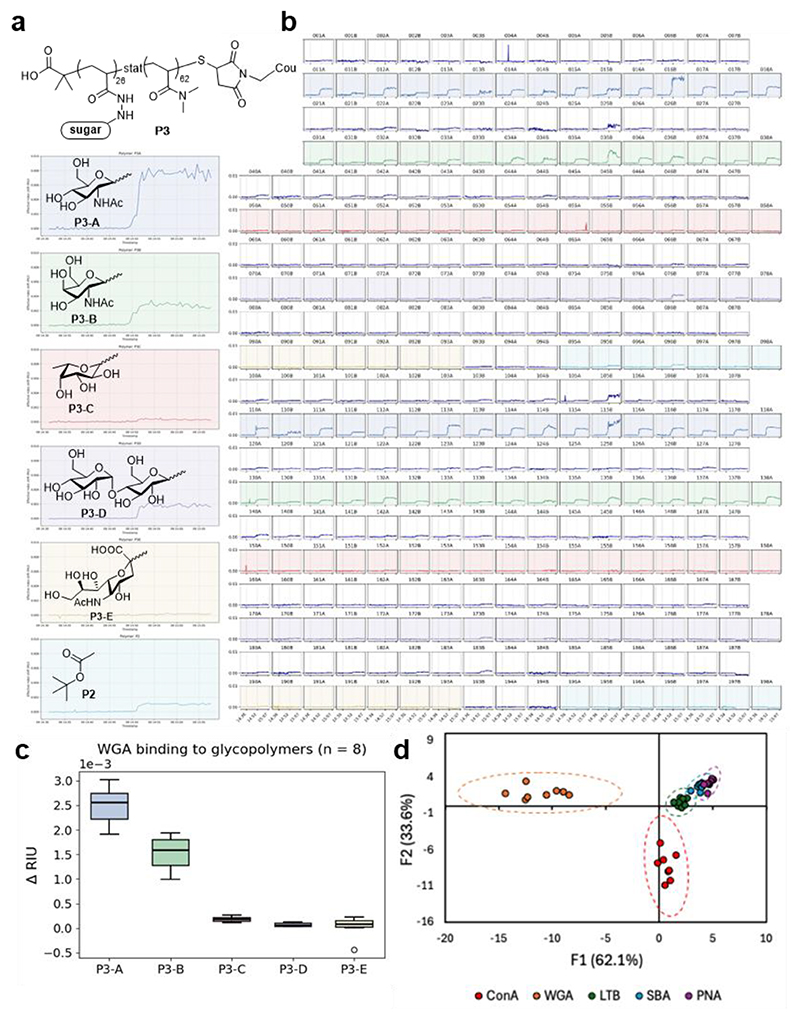
Array binding measurements: (a) Individual sensor responses of WGA binding to 5 glycopolymers (P3-A, -B, -C, -D, -E) and 1 control polymer (P2). (b) Full cGMR array response map of WGA binding to the 5 glycopolymers (colour coded corresponding to Fig. 3a), white sensors are control sensors functionalised with blocker only. (c) Box and whisker plots of the ΔRIU for the polymers P3-A to P3-E upon WGA addition, 8 replicates for each glycopolymer were chosen randomly from the full dataset to ensure equal weighting. (d) Canonical LDA score plots for analysis of a selection of lectin binding. The pairing of the first (F1) and second (F2) factors is shown. Dashed lines indicate 95% confidence intervals.

To demonstrate multiplexing, we functionalised the array with a library of five synthetic glycopolymers (P3-A–E) and a backbone control (P2) to map carbohydrate recognition patterns (Fig. 3a). These interactions, central to infection and cancer biology, are often difficult to characterise due to their weak, multivalent nature. Our platform overcomes this by capturing the full distribution of binding kinetics across 322 sensor pairs (glycopolymer synthesis, surface optimisation, and spotting protocols in SI S2, S4). The array was exposed to five lectins under flow: wheat germ agglutinin (WGA), concanavalin A (ConA), *E. coli* heat-labile toxin (LTB), soybean agglutinin (SBA), and peanut agglutinin (PNA). Individual sensor responses for WGA binding (Fig. 3b) and corresponding box-and-whisker plots (Fig. 3c) reveal inter-sensor heterogeneity, highlighting the necessity of array-resolved measurements for characterising complex, multivalent interactions. A concentration-dependent response was further confirmed through a dose-response analysis of WGA binding to P3-A compared to the P2 control (SI S5.3.2). From these measurements, we determined an LOD of 84 nM, indicating the sensitivity of the platform for detecting lectin-glycopolymer interactions.

The array yielded distinct carbohydrate-binding profiles for each lectin, which were analysed using Linear Discriminant Analysis (LDA) to classify the analytes (Stewart *et al*. 2014). This statistical approach reduces multivariate sensor responses into discriminant functions, creating a predictive model for lectin identification. Applying LDA to the array data enabled effective and unambiguous classification (Fig. 3d). Internal validation via a ‘leave-one-out’ cross-validation, where each datapoint is systematically excluded and reclassified, yielded 100% accuracy. To further assess the model’s robustness, we performed a hold-out validation study using 25 training points and 15 unknown samples; the LDA model correctly identified all unknown analytes with 100% confidence (SI S2.3). These results were corroborated by analogous in-solution fluorescence experiments (see SI for full details).

### 2.4 Portable Readout of cGMR Sensor Arrays

**Figure 4:**
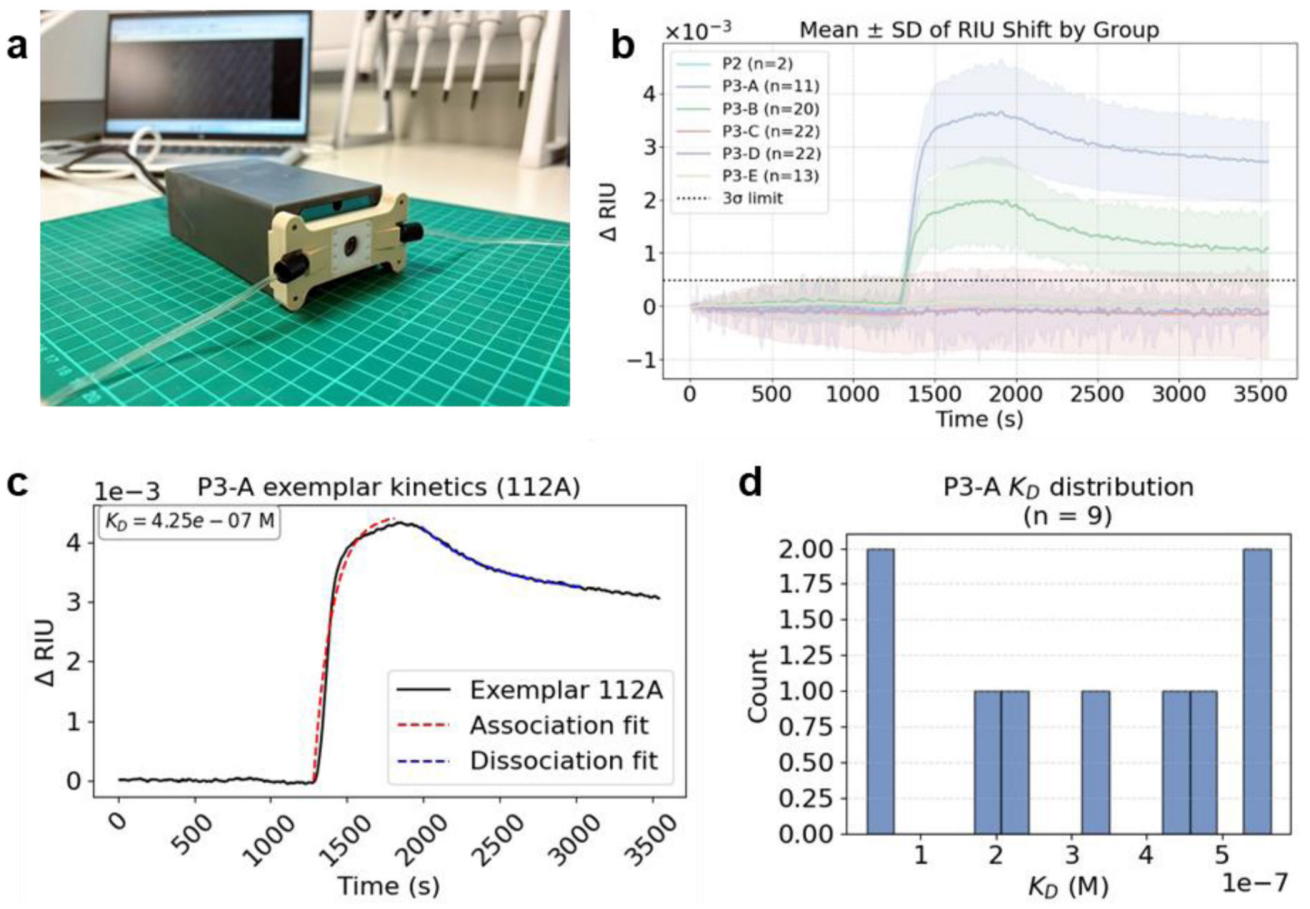
Portable array characterisation: (a) Photograph of portable optical setup (*Optical setup 3*, section 5.3) with flowcell attached. (b) Mean GMR response of WGA binding grouped by glycopolymer recorded on the portable setup. (c) Binding kinetics for WGA binding to P3-A on a single cGMR pair (sensor 112A): apparent *K_D_* determined using two-phase exponential model. (d) Distribution of apparent P3-A *K*_D_ values from 9 independent cGMR pairs measured in parallel.

To show the potential for operation of the array outside the laboratory and demonstrate the unique portability advantage of our approach, we performed measurements with a compact optical readout instrument (Fig. 4a). The instrument is an existing miniaturised version of the laboratory setup and is based on a 4.5 × 6 × 3 cm CNC-machined aluminium chassis housed in a bespoke casing (Gilroy *et al*. 2025, Drayton *et al*. 2020). This configuration of optical components can capture 185 differential sensor pairs within a single field of view. In the present laboratory experiments, fluid delivery was controlled using a peristaltic pump however, passive microfluidic cartridges for sample handling, as previously demonstrated for GMR-based detection (Gilroy *et al*. 2025), would allow a fully portable platform.

Despite the simplicity, resonance shifts for the glycopolymer-lectin interactions were stably tracked across the array and sensor responses were comparable to those obtained using benchtop instrumentation (Fig. 4b). The temporal resolution achieved with the portable setup was sufficient to resolve both association and dissociation phases of binding, enabling extraction of apparent kinetic rate constants (*k*_on_ and *k*_off_) and equilibrium dissociation constants (*K*_D_) from array-resolved measurements (Fig. 4c). Here, the binding kinetics were analysed using a two-phase exponential model, capturing fast and slow binding components during both association and dissociation. While a phenomenological approximation, this approach accounts for the heterogeneous and potentially multivalent nature of glycopolymer–lectin interactions and provides a robust framework for comparing effective binding kinetics across sensors in the array. For WGA binding to the P3-A synthetic glycopolymer, the mean apparent *K*_D_ measured for a single cGMR pair in the array (336 nM) is lower than literature values for WGA binding to a monovalent (GlcNAc)_2_ oligosaccharide (165 μM) (Lienemann *et al*. 2009). This difference is consistent with avidity effects arising from the multivalent display of GlcNAc motifs on the synthetic glycopolymer (Lundquist *et al*. 2002). Critically, rather than yielding a single apparent affinity, array-resolved measurements on the portable cGMR device allow us to reveal distributions of apparent equilibrium dissociation constants across multiple independent sensors. For WGA binding to P3-A, *K*_D_ values were found to range between 28 and 564 nM (median 336 nM, n = 9) (Fig. 4d). Importantly, this is consistent with the value of *K*_D_ estimated from the independent dose-response curve (*K*_D_ ≈ 253 nM, SI S5.3.1), providing further validation of the array’s quantitative performance. Independent measurements of WGA spiked into human plasma samples were also performed (SI S5.3) to evaluate the future clinical performance of the biosensor array. Although a significant non-specific response due to fouling was observed, this can be corrected through the inclusion of additional control sensors within the array, enabling accurate detection of lectin– glycopolymer interactions in a clinically relevant matrix. These results demonstrate that fully parallel interrogation, high temporal resolution, and differential sensing are preserved under portable operating conditions, enabling quantitative, kinetic biosensing with a compact, robust nanophotonic readout. Moreover, the ability to perform array-format measurements on a portable platform not only allows multiplexed detection but also provides robust, statistically validated kinetic parameters in a single experiment, even in clinical matrices and exposes outlier responses that would be invisible in conventional single- or few-channel measurements.

## 3 Discussion

The reported nanophotonic array provides a scalable, label-free platform integrating 322 differential sensors to capture real-time binding across hundreds of parallel interactions. CMOS-compatible fabrication ensures high reproducibility, with sensor-to-sensor optical variation below 5%. The multiplexed platform not only allows simultaneous detection of multiple analytes but also reveals statistically significant variability in surface functionalisation and molecular binding which are unresolved by singleplex portable photonic platforms. A key strength is the platform’s multidimensionality; diverse biorecognition molecules (antibodies, aptamers, and glycopolymers) can be probed simultaneously and with high temporal resolution to extract kinetic parameters (*k*_on_, *k*_off_, and *K*_D_). Crucially, our array-resolved analysis moves beyond mean affinity values to measure the actual distribution of *K*_D_ values across identical sensing elements. For WGA binding to P3-A glycopolymers, this distribution reflects surface heterogeneity and multivalent interactions while remaining clustered in the sub-micromolar range. Such distribution-level data, inaccessible to single-sensor measurements, provides an internal metric for data quality and surface uniformity which are critical for reproducible biomedical assays.

Unlike prism-based SPRi or ring-resonator systems, the cGMR architecture is intrinsically compatible with simplified, portable readout. Spatially encoding spectral information within chirped gratings allows for plane-wave illumination and camera-based imaging, eliminating fiber coupling or scanning optics. This design shifts the performance burden from complex optical hardware to the nanoscale device itself. A detailed comparison of the performance metrics against established SPRi and Genalyte platforms is provided in Table S17 (See SI).This work builds on demonstrated cGMR capabilities, including 1 pg/mL protein detection (Kenaan et al. 2020), operation in wound fluid, urine, and plasma (Bakshi *et al*. 2023, 2025; Gilroy *et al*. 2025), and handheld implementation (Drayton *et al*. 2020; Li *et al*. 2024). By retaining these capabilities at high sensor density with a simplified readout, this platform addresses the growing demand for accurate, personalised companion diagnostics (MarketsandMarkets, 2024).

## 4 Conclusion and Outlook

By combining nanoscale optical resonances with massively parallel, alignment-insensitive interrogation, the cGMR array provides a powerful tool for dissecting multivalent and heterogeneous interactions. The platform is designed to be scalable; the number of sensors can be increased by expanding the array with cross-talk minimised through chip design, and the analysis of each sensor is fully parallelisable. Our current instrument has the capacity to process hundreds of sensors in seconds (~6 s to process each image of the full 322 sensors, ~20 ms per sensor), and future implementations can leverage multi-core or distributed computing to accommodate even larger arrays, preserving both temporal resolution and data fidelity. This platform opens opportunities for large-scale molecular discovery, quantitative assay development, and supports the development of more informative measurement strategies relevant to emerging healthcare models such as personalised diagnostics.

## 5 Methods

### 5.1 Chip-scale GMR Array

The chips were fabricated by the Interuniversity Microelectronics Centre (imec) on a quartz substrate covered by a 150 nm silicon-nitride layer. Grating structures were defined by 193 nm DUV lithography at imec, and the devices were thereafter conformally coated with a 10 nm aluminium-oxide layer to enhance chemical resistance. The array chip comprises 322 pairs of gratings, with each individual grating measuring 250 × 100 μm. Two grating types are included on each chip: one chirped from a period of 420 nm to 424 nm, and the other from 421 nm to 425 nm. This configuration provides a spectral overlap between grating types, leading to a broadening of the effective dynamic range of the array.

### 5.2 Materials

All chemicals and solvents were used as supplied unless otherwise detailed. Acetone, dopamine HCl, Tris buffer, phosphate buffer saline (PBS), WGA, ConA, PNA, and SBA were purchased from Sigma Aldrich. Anti-CRP and CRP were purchased from Scripps Laboratories. SuperBlock™ was purchased from Thermofisher. Lectin LTB was expressed and purified; details are provided in the SI section S2.2. The synthesis of the glycopolymers (P3-A, P3-B, P3-C, P3-D, and P3-E) as well as the control polymer (P2) is provided in the SI.

### 5.3 Optical Setups

*Optical Setup 1*, the optical setup used to characterise the sensor, includes a collimated white light source (*ASBN-W HighPower Light Source*) and a monochromator (*Digikröm CM110 Monochromator*), with a 2.8 MP *camera* (*CoolSNAP DYNO*), in a reflection measurement configuration. At 4X magnification, the field of view on this setup captured 77 sensor pairs, 154 individual GMR sensors. The 4 nm range of one cGMR is 182 px on *Optical Setup 1*. Data in Figure 1 and 2 were collected using this setup.

*Optical Setup 2*, the second optical setup used to measure the full array, includes a collimated resonant cavity LED (RCLED) with wavelength centred at 650 nm (Roithner Lasertechnik - RC-LED-650-02), a film polariser (Thorlabs - LPVISE2X2) and a 1 nm FWHM bandpass filter centred at 647.1 nm (Andover - 647FS02-12.5)), with a 64 MP camera (Pi Hawk-eye), in a reflection measurement configuration. The field of view on this setup captured all 322 sensor pairs, 644 individual GMR sensors. The 4 nm range of one cGMR is 354 px on *Optical Setup 2*. Data in Figure 3 were collected using this setup.

*Optical Setup 3*, the portable optical setup, is a miniaturised version of *Optical setup 2* mounted in a 4.5 x 6 x 3 cm, CNC-machined aluminium chassis inside a 7 x 12 x 4 cm 3D-printed case. The instrument includes almost identical components to *Optical Setup 2*; a collimated RCLED with wavelength centred at 650 nm (Roithner Lasertechnik - RC-LED-650-02), a film polariser (Thorlabs - LPVISE2X2) and a 1 nm FWHM bandpass filter centred at 647.1 nm (Andover - 647FS02-12.5)), with a 12 MP camera (The Imaging Source - DMM 37UX226-ML), in a reflection measurement configuration. The field of view on this setup captured 185 sensor pairs, 370 individual GMR sensors. The 4 nm range of one cGMR is 255 px on *Optical Setup 3*. Data in Figure 4 were collected using this setup.

### 5.4 GMR Running Protocol

The flow cell (Fig. S19) was initially filled with the relevant starting solution (water or buffer, see specific experiments for details) then flowed for ≥10 minutes to provide a baseline reading. Flow was provided by a syringe pump, pulling at 100 mL min^-1^, creating flow in the direction of right to left across the field of view. Test solutions were then introduced at the concentrations and for the timescales detailed in the results section, the flow rate of 100 mL min^-1^ was maintained throughout, pausing briefly to change tubing to the next solution. Following the test solution, buffer then flowed over the sensor again for ≥10 minutes. All experiments were carried out at 21 °C. Working solution concentrations were as follows: CRP antibody 50 mg mL^-1^ in flow, 250 mg mL^-1^ when spotted; SuperBlock™ x10 dilution of commercial stock in PBS; CRP antigen 20 mg mL^-1^; glycopolymers 250 mg mL^-1^; lectins 50 mg mL^-1^.

### 5.5 Image Analysis and Data Processing

Image analysis was performed using computer vision techniques to identify the array labels and determine the precise locations of the individual array cGMRs. The image was then partitioned into multiple regions of interest (ROIs), with two ROIs assigned per sensor (cGMR pair). Each ROI was processed by fitting a Gaussian function to each pixel row. Although the grating’s resonant response exhibits a Fano spectral shape - arising from the interaction between Fabry–Perot and waveguide modes - a Gaussian function was used as a computationally efficient approximation. This procedure yielded approximately 150 Gaussian fits per ROI. The median Gaussian centre position (x_med_, in pixels) was taken as the resonant bar position, as the median is more robust to outliers than the mean. The interquartile range (IQR) of these positions was also computed to assess the quality of the resonant bar. Because the refractive index shift (ΔRIU) is directly related to the resonant position, the shift of the two resonant bars was calculated then the value in pixels was converted into a shift in resonant wavelength.

### 5.6 Discrimination of Lectin Binding using LDA Analysis

The raw data obtained from binding studies between the glycopolymer array and the selected lectins was processed by taking the mean value (20 data points) before and the mean value (20 data points) after the binding interaction and calculating the differential to gain the resonant shift in pixels (Table S5). These resonant shift values were subjected to LDA in SPSS (IBM) to develop the model (Table S6). LDA enabled effective discrimination of the analytes (Fig. 3d) and identification of analytes was achieved with 100% accuracy. Raw data and LDA can be found in the SI section S2.3 as well as a validation study mapping the glycopolymer-lectin interactions in solution using a fluorescence binding assay.

## Supplementary Material

Supporting Information

## Data Availability

The authors declare that the data supporting the findings of this study are available within the paper and the Supplementary Information files. Should any raw data files be needed in another format they are available from the corresponding authors upon request.

## Highlights

- CMOS compatible nanophotonic array enables massively parallel label-free biosensing
- Array-level readout resolves kinetic distributions masked by single-sensors
- Portable instrument delivers real-time analysis of diverse bioreceptors
