# Supplementary material for: Multiplexed Nanophotonic Sensor Arrays for Time-resolved Biomolecular Analysis
Source: Biosens Bioelectron. Author manuscript; Available in PMC 2026 Apr 22. (PMC7619026; doi:10.1016/j.bios.2026.118669)
Supplement: Supporting Information [file EMS213338-supplement-Supporting_Information.pdf]

Supporting Information

Multiplexed Nanophotonic Sensor Arrays for Time-resolved Biomolecular Analysis

Lisa M. Miller[1*], Christopher P. Reardon[1,2], Kathryn G. Leslie[3], Callum D. Silver[1,2], Joshua S. Male[1,2], Clare S. Mahon[3], Thomas F. Krauss[1,2] and Steven Johnson[1,2*]

[1]School of Physics and Technology, University of York, Heslington, York, YO10 5DD, UK
[2]Phorest Diagnostics Ltd., School of Physics and Technology, University of York, Heslington, York, YO10 5DD, UK
[3]Department of Chemistry, Durham University, Durham DH1 3LE, UK
*email: lisa.miller@york.ac.uk; steven.johnson@york.ac.uk

Contents

S1. Photonic Array Characterisation
- **1.1 Sensor Preparation**
- **1.2 Bulk Sensitivity**
- **1.3 Surface Functionalisation for Binding Studies**
- **1.4 RIU Shift to Surface Mass Density Calibration**
- **1.5 Quantification of Binding Densities**
- **1.6 CRP Binding Kinetics**

S2. Glycopolymers and Lectins
- **S2.1 Synthesis and Characterisation of Glycopolymers**
- **S2.2 Expression and Purification of LTB**
- **S2.3 Discrimination of Model Lectin Library**
- **S2.4 QCMD Data Confirming Glycopolymer Surface Chemistries**
  - **S2.4.1 QCMD: Instrumentation and Materials**
  - **S2.4.2 QCMD: Glycopolymer Blocker Optimisation**
  - **S2.4.3 QCMD: Glycopolymer Surface Characterisation**

S3. Flow Cell Details

S4. Spotting Optimisation

S5. Different Binders and Sample Matrices on the Array
- **S5.1 Antibody**
- **S5.2 Aptamers**
- **S5.3 Glycopolymers**
  - **S5.3.1 WGA Binding to P3-A Dose Curve**
  - **S5.3.2 Measurement in Human Plasma**

S6. Comparison of the cGMR array with Established Platforms

S7. References

S1. Photonic Array Characterisation

S1.1. Sensor Preparation

Sensors were initially cleaned by sonication (Fisherbrand™ S-series Ultrasonic Cleaner, FB15050.) in acetone (1 hour) followed by drying with N_2 then treatment by UV-ozone (1 hour, 5 sccm O_2, 100% power, Henniker Plasma UV-ozone plasma treatment system HPT-100). Cleaned sensors were measured in *Optical Setup 1* to identify the working wavelength range. For working in optical setups with a fixed wavelength, the working range was then tuned to centre around 647 nm by atomic layer deposition (ALD) coating with TiO_2 (1 nm TiO_2 coating provides +1.3 nm wavelength shift). ALD coating of TiO_2 was carried out using tetrakis(dimethylamido)titanium (TDMAT) and water precursors, deposited at 100 dC and 90 dC respectively (Picosun R-200 Standard ALD). The TDMAT precursor growth rate was 0.06 nm/cycle. Prior to SEM imaging, sensors were spin-coated in AR-PC 5090 (allresist), at 2000 rpm 60 seconds, 90 dC 2 mins (Electronic Micro Systems EMS 4000). SEM images were captured using a JEOL SEM JSM-7800F.

S1.2. Bulk Sensitivity:

The cGMR sensor array was fitted into a bespoke fluidic cell which allowed a series of ethanol/water solutions (0%, 1%, 2%, 4%, 8% corresponding to a bulk refractive index (RI) of 1.3330, 1.3336, 1.3342, 1.3351, 1.3360, respectively) to be flowed over the photonic array while the shift in resonance was monitored (Fig. S1a). From this data the bulk RI sensitivity (Fig. S1b and c) and the systemic measurement noise (Fig. S1d and e) were determined.

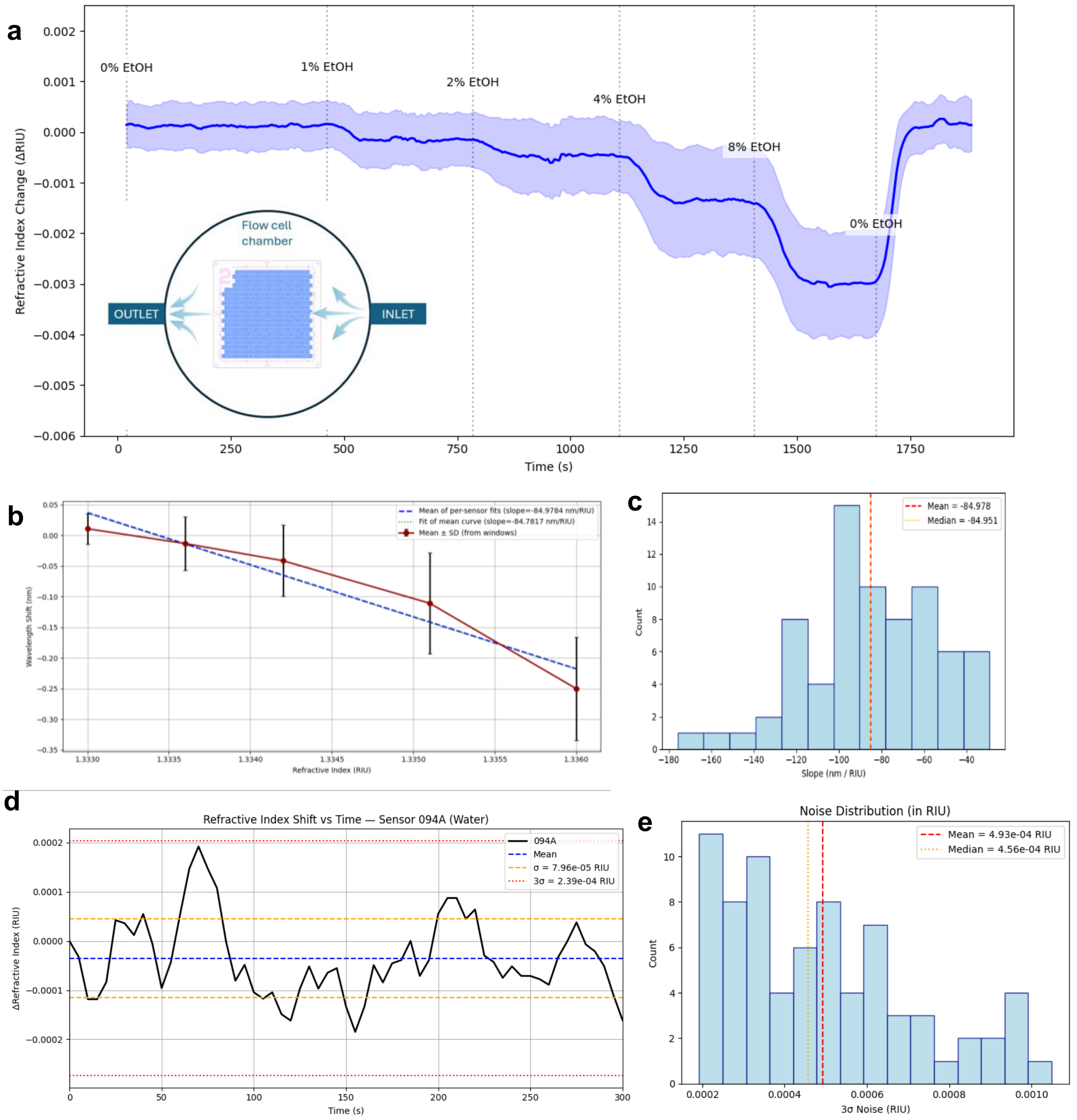

Figure S1: Bulk sensitivity measurement on *Optical Setup 1*: (a) Resonance wavelength shift versus time as ethanol solutions were flowed over, inset diagram of flow cell (b) Average resonance wavelength shift (for n = 79 cGMR sensor pairs) versus RIU. An average bulk sensitivity of 85 nm/RIU is calculated from a line fit to these data; (c) Inter sensor distribution of bulk sensitivity with the mean (red) and median (yellow) indicated; (d) Wavelength shift versus time for a sensor exposed to ultrapure water over 5 minutes, exemplar shown for one sensor (sensor number 094A). The blue, yellow, and red lines indicate mean, one standard deviations (σ), and 3σ respectively; (e) Inter sensor distribution of 3σ values with the mean (red) and median (yellow) indicated.

The measured median sensitivity of the miniaturised cGMRs in the array (85.0 nm/RIU) is comparable to that of our prior larger cGMR (88.3 nm/RIU, Fig. S2), when characterised on *Optical Setup 1*. With a sensitivity of 85.0 nm/RIU, the 5 nm working range of the photonic array is equivalent to 0.06 RIU, well suited to measuring thin biomolecular layers with typical magnitudes of 10^{-6}-10^{-3} RIU. The smallest detectable resonance shift (the limit of detection), determined from 3 times the standard deviation (σ) in RIU shift for water, was determined by measuring the noise over 5 min (Fig. S1d) for each sensor in the array. The median 3σ of the array was calculated to be 4.56×10^{-4} RIU (Fig. S1e).

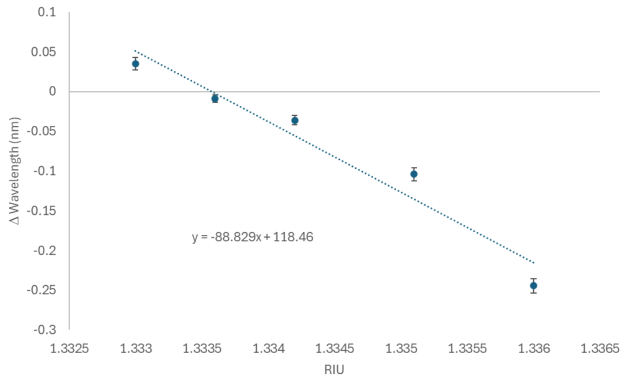

Figure S2: Sensitivity of larger cGMR: Resonance wavelength shift versus RIU (8 sensors). Mean values (±sd).

S1.3. Surface Functionalisation for Binding Studies

The sensor was first cleaned by UV-ozone (10 min, 5 sccm O_2, 100% power) then coated with a layer of PDA by submersion in a solution of dopamine HCl (2 mg mL^{-1} in 5 mM Tris buffer pH 8.5) for 15 minutes. Sensors were held vertically during PDA formation to avoid any aggregates in the solution settling on the sensor surface. The sensor was then washed with ultra-pure water and dried with N_2. For functionalisation of the binder molecules by spotting, PDA-coated sensors were then loaded on the stage of an automated piezo-driven, non-contact spotter (SCIENION sciFLEXARRAYER-S3) fitted with a piezo dispense capillary (PDC) 100 (spotting volume 520-600 pL), with type 2 coating for spotting protein mixtures. Solutions were spotted at a concentration of 250 μg mL^{-1}. Once spotted with the required binder, sensors were incubated at ambient temperature 15 minutes inside the humidity chamber of the spotter to allow for functionalisation (for optimisation of spotting protocols see SI). Antifouling blocking was then carried out using SuperBlock™ (commercial stock was first diluted ×10 using PBS), which was pipetted on top of the spotted sensor and allowed to incubate for a further 15 minutes. The functionalised sensor was then gently rinsed with ultra-pure water, dried with N_2 and loaded into the flow cell (Fig. S19) for testing.

S1.4. RIU shift to surface mass density calibration

QCMD data shows 52.6352 ng/cm^2 mass change for 50 μg/mL anti CRP binding to PDA layer formed on an SiO2 QCMD sensor. cGMR shows a 44.8×10^{-4} RIU ($\pm 7.3 \times 10^{-5}$) shift for this same step. Mass bound to the QCMD sensor surface was calculated using the frequency shift from the 7th harmonic (Fig. S3) with the Sauerbrey equation [1]. We accept that this method of analysis assumes a thin, rigid, and uniform layer, and is therefore only an estimated value.

From this we determined a conversion factor of **1×10^{-4} RIU shift = 1.17489 ng/cm^2**

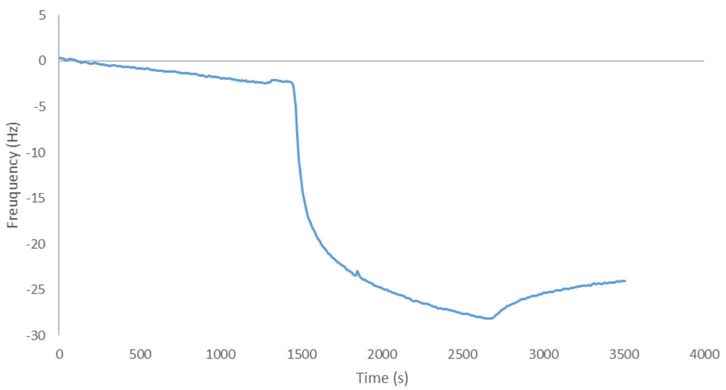

Figure S3: QCMD measurement of 50 µg/mL anti CRP binding to PDA functionalised sensor, 7th harmonic plotted. Frequency shift -20.8162 Hz.

S1.5. Quantification of binding densities

The surface concentration of surface-bound molecules can be calculated from the measured change in RI (Table S1), the cGMR surface area (**5.53×10^{-4} cm²**; see below for calculation), the average sensor sensitivity (**85.0 nm/RIU**) and the mass conversion factor (**1×10^{-4} RIU shift = 1.17 ng/cm²**).

Surface area calculation

cGMR sensor area: 250 µm × 100 µm = 2.5×10^{-4} cm² (footprint)

Grating topography: chirped period (420-425 nm) and the etch depth (150 nm). Using an average period of 422.5 nm and the depth of 150 nm, the surface area of the 250 µm × 100 µm footprint can be calculated to be:

$$A_{total} = A_{top} + A_{sidewalls} + A_{bottom}$$
$$= 25000 + 17760 + 12530$$
$$= 5.53 \times 10^{-4}\ \text{cm}^2$$

Table S1: Measured ΔRIU mean

Step	Binder	ΔRIU mean (± s.d.)
**1**	Anti-CRP	44.7×10^{-4} ($\pm 7.3 \times 10^{-4}$)
**2**	SuperBlock™	3.5×10^{-4} ($\pm 1.0 \times 10^{-4}$)
**3**	CRP	9.7×10^{-4} ($\pm 2.1 \times 10^{-4}$)

Step 1 - antibody binding to PDA

Protein: anti-CRP, MW = 150 kDa; Shift in RIU = 44.7×10^{-4}

Total per sensor:

mass = $\Gamma \cdot A = (44.7 \cdot 1.17\ \text{ng/cm}^2) \cdot 0.000553\ \text{cm}^2 = 0.029$ ng

n moles = 0.029×10^{-9} g / 150,000 g/mol = 1.9×10^{-16} mol

n molecules = $1.9 \times 10^{-16} \cdot 6.022 \times 10^{23} = 11.4 \times 10^{7}$ molecules

Density of anti-CRP: $11.4 \times 10^{7} / 5.53 \times 10^{-4}$ cm² = 2.06×10^{11} molecules/cm²

Step 3 - antigen binding to antibody

Protein: CRP, MW = 115 kDa; Shift in RIU = 9.7×10^{-4} ($\pm 2.1\times10^{-4}$)

Total per sensor:

mass = $\Gamma \cdot A$ = (9.7·1.17 ng/cm²)·0.000553 cm² = 0.0006 ng

n moles = 0.0006×10^{-9} g / 115,000 g/mol = 5.46×10^{-17} mol

n molecules = $5.46 \times 10^{-17} \cdot 6.022 \times 10^{23}$ = 3.29×10^{7} molecules

Density of CRP: $3.29 \times 10^{7} / 5.53 \times 10^{-4}$ cm² = 5.94×10^{10} molecules/cm²

S1.6. CRP Binding Kinetics

To enable quantitative comparison between functionalisation and binding steps, the temporal evolution of the sensor signal was fitted using a single-exponential function describing the approach to steady state (Fig. S4). This empirical model was used only to extract characteristic response rates and does not represent a mechanistic binding model of the underlying surface processes.

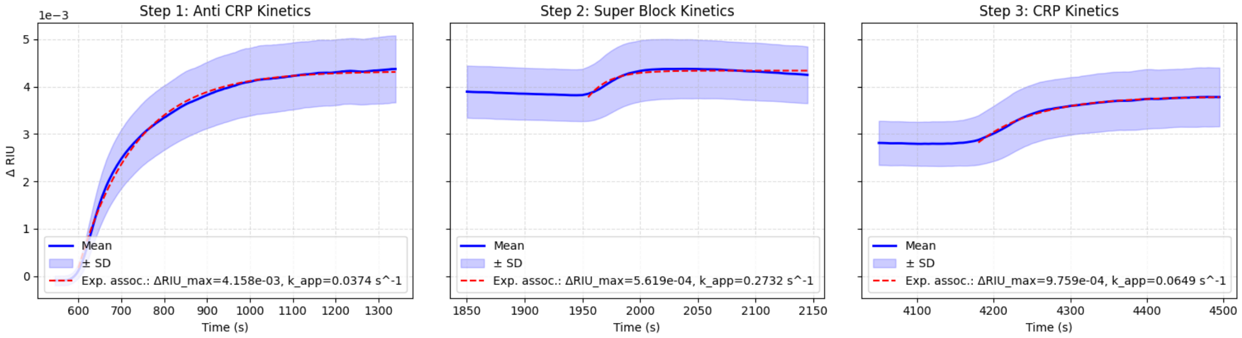

Figure S4. Binding kinetics for each stage in the surface functionalisation process: antibody binding to CRP, superblock and CRP-antibody binding: apparent on-rate (k_app) and maximum RIU shift (ΔRIU) determined using the first-order exponential fit to the mean data (79 cGMR sensor pairs).

S2. Glycopolymers and Lectins
S2.1 Synthesis and Characterisation of Glycopolymers

General Experimental Details

All reagents were purchased from Sigma Aldrich, Fischer Scientific, Biosynth or Fluorochem and used as received unless otherwise stated. *N*,*N*-dimethylacrylamide was passed through basic Al_2O_3 immediately prior to use. Dialysis membrane (regenerated cellulose with a 3500 molecular weight cut-off) was obtained from Spectrum Dialysis. RAFT chain transfer agent S-dodecyl-S′-(α,α′-dimethyl-α″-acetic acid)trithiocarbonate DDMAT was synthesised according to a literature method.[5]

Instrument and analysis

NMR spectra were recorded on a Bruker DRX-400 MHz spectrometer (at operating frequencies of 400.13 MHz for ^{1}H, 100.62 MHz for ^{13}C) using commercially available deuterated solvents ($CDCl_3$ (δH = 7.26 ppm), and D_2O (δH = 4.79 ppm)). Electrospray ionisation mass spectra (ESI-MS) were obtained on a TQD mass spectrometer with an Acquity UPLC and Acquity photodiode array detector for absorbance data; acetonitrile was used as the solvent. Gel permeation chromatography measurements were conducted using an Agilent 1260 instrument equipped with differential refractive index detector and a pair of PL gel 5 µm Mixed-D columns (300 × 7.5 mm) with a guard column (Polymer Laboratories Inc.), connected in series. Chromatography was performed in DMF with LiBr (1 g/L) at a flow rate of 0.6 mL/min at 50 °C. Near monodisperse poly(methyl methacrylate) standards (Agilent) were used for calibration. Ultraviolet-Visible (UV-Vis) and fluorescence spectra were obtained using a Tecan SPARK® multimode microplate reader.

Synthesis

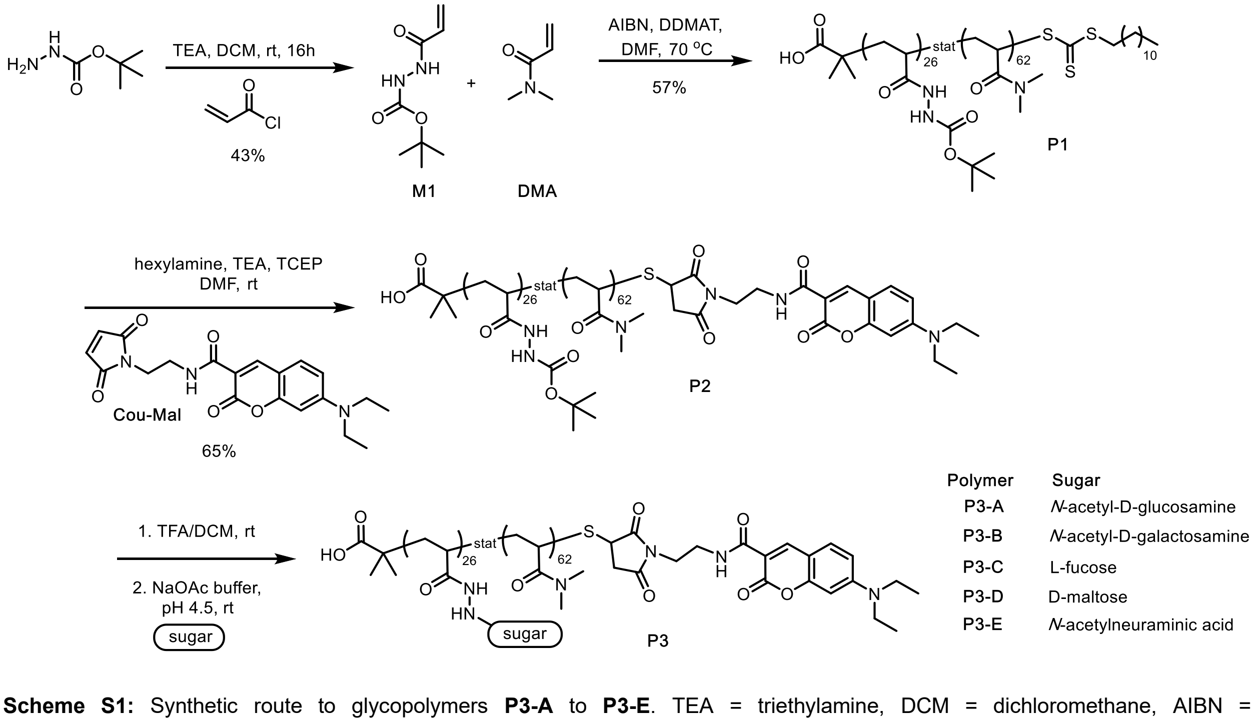

Scheme S1: Synthetic route to glycopolymers **P3-A** to **P3-E**. TEA = triethylamine, DCM = dichloromethane, AIBN = Azobisisobutyronitrile, DDMAT = S-dodecyl-S′-(α,α′-dimethyl-α″-acetic acid)trithiocarbonate, DMF = dimethylformamide, TCEP = (tris(2-carboxyethyl)phosphine), TFA = trifluoroacetic acid.

M1

Acryloyl chloride (4.46 mL, 55 mmol) in CH_2Cl_2 (20 mL) was added dropwise to a solution of *tert*-butyl carbazate (6.6 g, 50 mmol) and triethylamine (7.62 mL, 55 mmol) in CH_2Cl_2 (100 mL) at 0 ˚C. The reaction was warmed to room temperature and stirred for 24 h. The reaction mixture was then washed with water (50 mL × 2) and brine (50 mL), and dried over anhydrous sodium sulfate, before being evaporated to dryness. The crude residue was recrystallised in CH_2Cl_2 to give **M1** as white crystals (4.02 g, 43%) with spectra matching the literature.[6] **^{1}H NMR** (400 MHz, $CDCl_3$) δ 8.52 (sb, 1H), 7.02 (sb, 1H), 6.39 (dd, *J* = 17.0, 1.4 Hz, 1H), 6.18 (dd, *J* = 17.0, 10.4 Hz, 1H), 5.72 (dd, *J* = 10.4, 1.4 Hz, 1H), 1.46 (s, 9H); **^{13}C NMR** (101 MHz, $CDCl_3$) δ 165.0, 155.9, 128.7, 127.9, 82.2, 28.3; **ESI-MS** [M+H$^+$] 187.1

P1

S-1-Dodecyl-*S'*-(α,α-dimethyl-α''-acetic acid)trithiocarbonate (DDMAT) (30 mg, 0.08 mmol, 1.0 eq.), *α,α'*-azoisobutyronitrile (AIBN) (2.7 mg, 0.016 mmol, 0.2 eq.), *N,N*-dimethylacrylamide (**DMA**) (0.65 g, 6.58 mmol, 80 eq.), **M1** (0.61 g, 3.3 mmol, 20 eq.) and DMF (4.5 mL) were combined. $Ar_{(g)}$ was bubbled through the solution for 20 min, then the vessel was placed in a preheated oil bath at 70 °C. The reaction was monitored by ^{1}H NMR spectroscopy, and after 90 min (73% conversion) the polymerisation was quenched by rapid cooling in $N_{2(l)}$ followed by exposure to air. The solution was added dropwise to rapidly stirring Et_2O, and the solvent decanted. The crude precipitate was dissolved in methanol and dialysed against methanol (1:400 v:v ratio, 3 rounds). The methanol was evaporated and the product isolated by lyophilisation from dioxane to afford **P1** as a white solid (0.53 g, 57%). **^{1}H NMR** (400 MHz, $CDCl_3$): 1.10 – 1.45 (br, C(C*H$_3$)$_3$*), 1.46 – 1.96 (br, C*H*CH$_2$), 1.97 – 2.46 (br, CHC*H$_2$*), 2.49 – 3.20 (br, N(C*H$_3$)$_2$*), 6.25 – 8.0 (br, N*H*-NH), 8.34 – 10.00 (br, NH-N*H*).

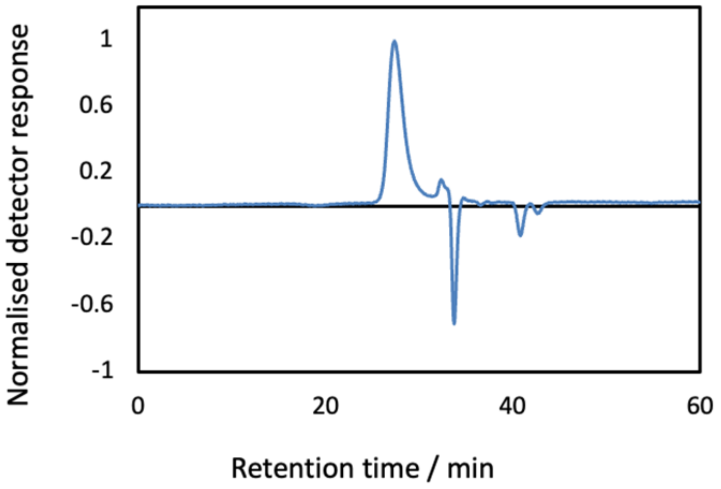

Figure S5: Differential refractive index gel permeation chromatogram of **P1**, acquired in DMF with LiBr (1 g/L) at a flow rate of 0.6 mL/min at 50 °C.

Table S3: Characteristics of **P1**. [a] As determined by conversion analysis using ^{1}H NMR spectroscopy. [b] As determined by gel permeation chromatography in DMF with LiBr (1 g/L).

Polymer	DMA (equiv.)	DMA Conv. (%)	DMA units	**M1** (equiv.)	**M1** conv. (%)	**M1** units	*M*n^a (g mol^{-1})	*M*n^b (g mol^{-1})	*M*w^b (g mol^{-1})	Ð *M*w/*M*n
**P1**	80	77	62	40	65	26	11,300	10,900	14,000	1.28

P2

P1 (500 mg, 44 μmol), TCEP (25 mg, 88 μmol), and **Cou-Mal** (50.8 mg, 132 μmol) were dissolved in DMF (4 mL) and the solution sparged for 10 minutes with $Ar_{(g)}$. Triethylamine (12 μL, 88 μmol) and hexylamine (30 μL, 220 μmol) were added and the solution was sparged for a further 5 minutes, then left to stir for 16 h under an atmosphere of Ar. The crude reaction mixture was precipitated into Et_2O, and the solvent decanted. The crude precipitate was dissolved in methanol and dialysed against methanol (1:400 v:v ratio, 3 rounds, until the dialysate was no longer coloured). The methanol was evaporated and the product isolated by lyophilisation from dioxane to afford **P2** as a yellow solid (327 mg, 65%). **^{1}H NMR** (400 MHz, $CDCl_3$): 1.20 – 1.54 (br, C(C*H*$_3$)$_3$), 1.55 – 2.04 (br, C*H*CH$_2$), 2.05 – 2.76 (br, CHC*H*$_2$), 2.77 – 3.25 (br, N(C*H*$_3$)$_2$), 6.25 – 7.77 (br, N*H*-NH), 7.95 – 8.22 (br, m, coumarin), 8.29 – 10.18 (br, NH-N*H*); **UV-Vis** (HEPES buffer, pH 7.4) abs λ_{max} = 427 nm, ε = 21,220 M^{-1} cm^{-1}.

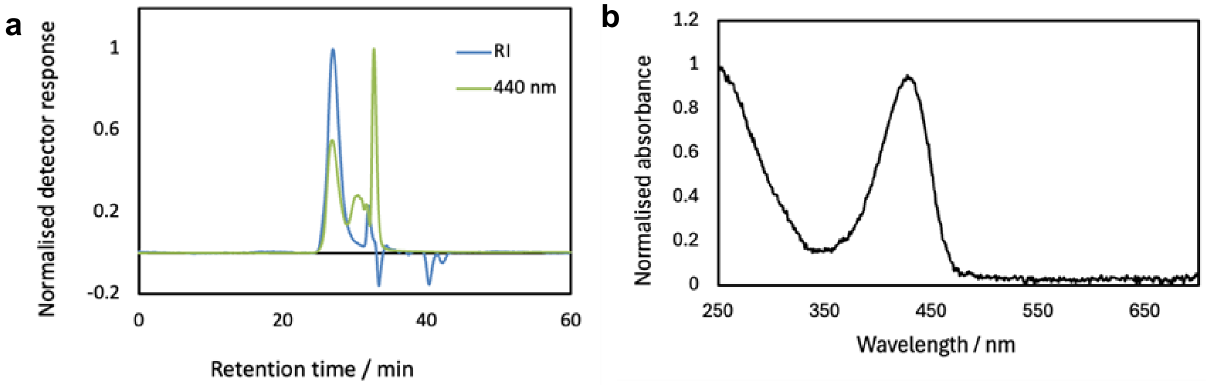

Figure S6: (a) Differential refractive index gel permeation chromatogram of **P2**, acquired in DMF with LiBr (1 g/L) at a flow rate of 0.6 mL/min at 50 °C, showing refractive index detector response and UV-Vis detector response at 440 nm. (b) Absorption spectrum of **P2** (5 μM) in HEPES buffer (pH 7.4).

P3 general method

P2 (50 mg, 4.5 μmol) was dissolved in 20% TFA in CH_2Cl_2 (2 mL) and stirred at rt for 2 h. The solvent was removed and the crude product was isolated by lyophilisation from deionised H_2O. This product was then dissolved in 0.1 M NaOAc buffer (2 mL, pH 4.5, 1 mM aniline additive) and corresponding reducing sugar (10 equiv. per side chain) and stirred at 40 ºC for 18 h. The reaction mixture was concentrated by centrifugation (3871 rcf, 25 min, M_w cutoff 3.5 kDa), diluted with deionised H_2O (10 mL) and concentrated twice more, before removing the solvent by lyophilisation to give **P3-A**, **B**, **C**, **D**, and **E** as pale yellow solids (Table S4, Figure S7, NMR spectra below).

Table S4: P3-sugar polymers **A – E** synthesis and characterisation details.

Polymer	Sugar	Sugar in reaction (mg)	Sugar in reaction (μmol)	Isolated polymer mass (mg)	Isolated polymer yield (%, 2 steps)	% sugar attachment (by NMR)	Polymer M_n
**P3-A**	GlcNAc	258	1170	32	65	80	13,300
**P3-B**	GalNAc	258	1170	34	68	80	13,300
**P3-C**	Fuc	192	1170	40	80	88	12,500
**P3-D**	Mal	427	1170	24	48	90	17,200
**P3-E**	Neu5Ac	361	1170	34	68	57	13,300

Polymer library

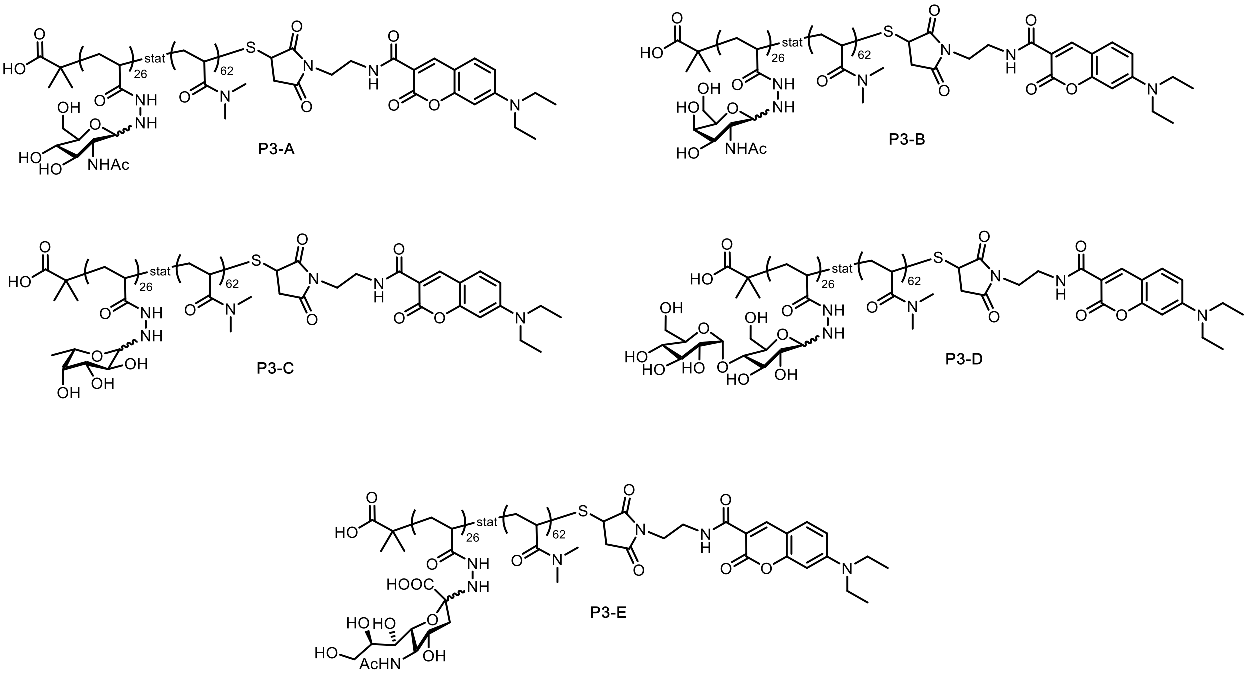

Figure S7: Structures of glycopolymers **P3-A** to **P3-E**.

Coumarin-maleimide synthesis

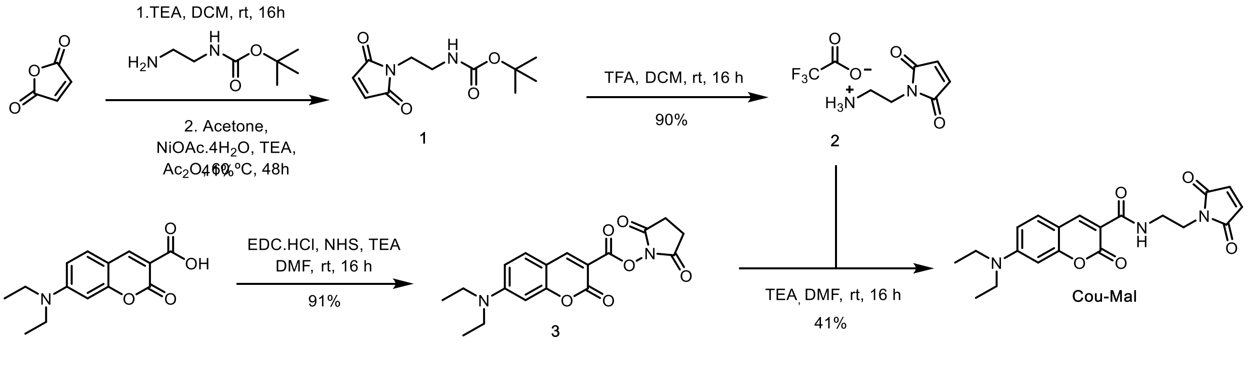

Scheme S2: Synthetic route to **Cou-Mal** for tagging polymer **P1**.

Boc-aminoethylene maleimide (1)

Maleic anhydride (4.5 g, 45.9 mmol) was dissolved in CH_2Cl_2 (90 mL) and cooled to 0 °C. *N*-Boc ethylene diamine (6.66 g, 41.5 mmol) was dissolved in anhydrous CH_2Cl_2 (90 mL) and added dropwise to the maleic anhydride solution and then stirred overnight at room temperature. The CH_2Cl_2 was then evaporated, and the white residue was dissolved in acetone (180 mL) giving a clear colourless solution. Nickel acetate (225 mg, 0.90 mmol) acetic anhydride (15.9 mL, 168 mmol) and triethylamine (2.4 mL, 17.2 mmol) were added to the solution and the mixture was heated to 65 °C for 2 days. After approximately 1 hour the solution had turned from a clear pale-yellow solution to black. After the reaction, the mixture was cooled to room temperature and the acetone removed giving a thick black liquor which was poured over ice water (200 mL) and stirred for 30 mins. A black precipitate was collected by sinter filtration and washed with H_2O (2 × 50

mL). This crude residue was dried and purified via silica flash chromatography (1:0 to 0:1 heptane:ethyl acetate) to give the product **1** as a white solid with spectra matching the literature[7] (4.04 g, 41%). **^{1}H NMR** (400 MHz, $CDCl_3$) δ 6.71 (s, 2H), 4.73 (s, 1H), 3.66 (t, *J* = 5.5 Hz, 3H), 3.33 (q, *J* = 5.8 Hz, 2H), 1.40 (s, 9H). **^{13}C NMR** (101 MHz, $CDCl_3$) δ 170.90, 156.03, 134.26, 79.56, 39.43, 38.07, 28.39. **ESI-MS**: $[M+Na]^+$ 263.2.

Aminoethylene maleimide TFA salt (2)

1 (3.18 g. 13.2 mmol) was dissolved in 20% trifluoroacetic acid in CH_2Cl_2 (20 mL) and stirred for 16 h. Following the reaction, CH_2Cl_2 was removed, and the resulting yellow oil precipitated into Et_2O. The precipitate was filtered to give the product as a white crystalline solid with spectra matching the literature[8] (3.01 g, 90%). **^{1}H NMR** (400 MHz, D_2O) δ 6.93 (s, 2H), 3.86 (t, *J* = 5.9 Hz, 2H), 3.25 (t, *J* = 5.9 Hz, 2H); **^{13}C NMR** (101 MHz, D_2O) δ 172.68, 134.74, 38.43, 35.07.

NHS-diethylamino coumarin ester (3)

A solution of 7-(diethylamino)coumarin-3-carboxylic acid (1.00 g, 3.83 mmol) and triethylamine (585 µL, 4.21 mmol) in DMF (3 mL) was added dropwise to a solution of *N*-hydroxysuccinimide (0.48 g, 4.21 mmol) and EDC.HCl (0.81 g, 4.21 mmol) in DMF (3 mL). The reaction was stirred at room temperature overnight, then poured into water (100 mL). The resulting precipitate was isolated by filtration and washed with water (50 mL), then dried to give the product as a yellow solid with spectra matching the literature[9] (1.24 g, 91%). **^{1}H NMR** (400 MHz, $CDCl_3$) δ 8.57 (d, *J* = 0.6 Hz, 1H), 7.37 (d, *J* = 9.0 Hz, 1H), 6.63 (dd, *J* = 9.0, 2.5 Hz, 1H), 6.45 (dd, *J* = 2.5, 0.7 Hz, 1H), 3.47 (q, *J* = 7.2 Hz, 4H), 2.88 (sb, 4H), 1.25 (t, *J* = 7.2 Hz, 6H); **^{13}C NMR** (101 MHz, $CDCl_3$) δ 169.6, 159.3, 159.0, 157.2, 154.3, 151.3, 132.1, 110.3, 107.8, 102.7, 96.9, 45.5, 25.8, 12.6. **ESI-MS** $[M+H^+]$ 359.2.

Coumarin maleimide (Cou-Mal)

A solution of maleimide amine TFA salt (**2**) (196 mg, 0.77 mmol) and triethylamine (0.2 mL, 1.4 mmol) in DMF (2 mL) was added dropwise to a solution of coumarin **3** (0.25 g, ~0.7 mmol) in DMF (2 mL) under $Ar_{(g)}$. The reaction was stirred at room temperature overnight, then poured over ice. The resulting precipitate was isolated by centrifugation and dried to give the product as a yellow powder with spectra matching the literature[10] (111 mg, 41%).**^{1}H NMR** (400 MHz, $CDCl_3$) δ 8.88 (sb, 1H), 8.66 (s, 1H), 7.41 (d, *J* = 9.0 Hz, 1H), 6.69 (s, 2H), 6.63 (dd, *J* = 9.0, 2.4 Hz, 1H), 6.47 (d, *J* = 2.4 Hz, 1H), 3.83 – 3.70 (m, 2H), 3.68 – 3.59 (m, 2H), 3.44 (q, *J* = 7.2 Hz, 4H), 1.23 (t, *J* = 7.2 Hz, 6H). **ESI-MS** $[M+H^+]$ 384.3.

NMR Spectra of polymers

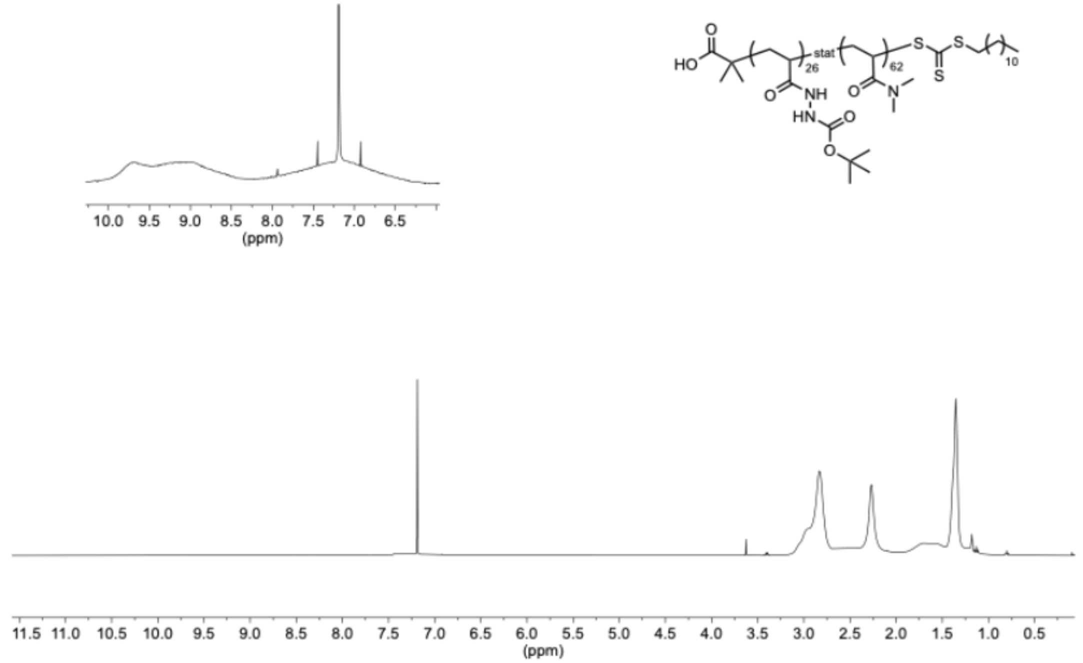

Figure S8: ^1H NMR spectrum (400 MHz, $CDCl_3$) of **P1**.

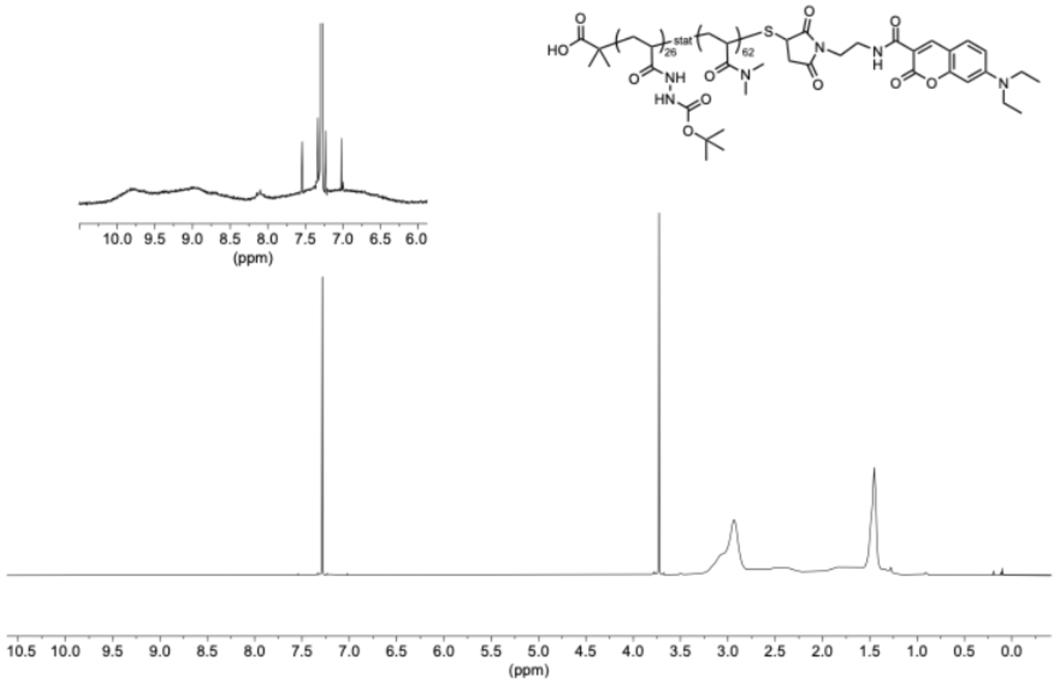

Figure S9: ^1H NMR spectrum (400 MHz, $CDCl_3$) of **P2**.

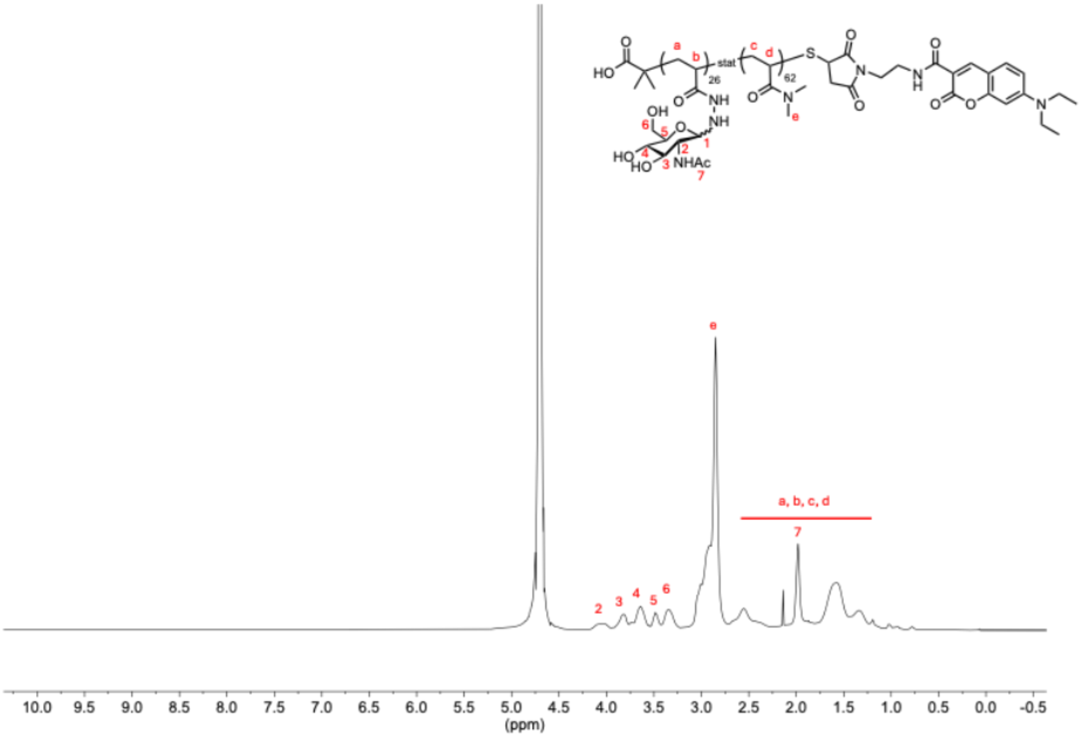

Figure S10: ${}^{1}H$ NMR spectrum (400 MHz, D_2O) of **P3-A**.

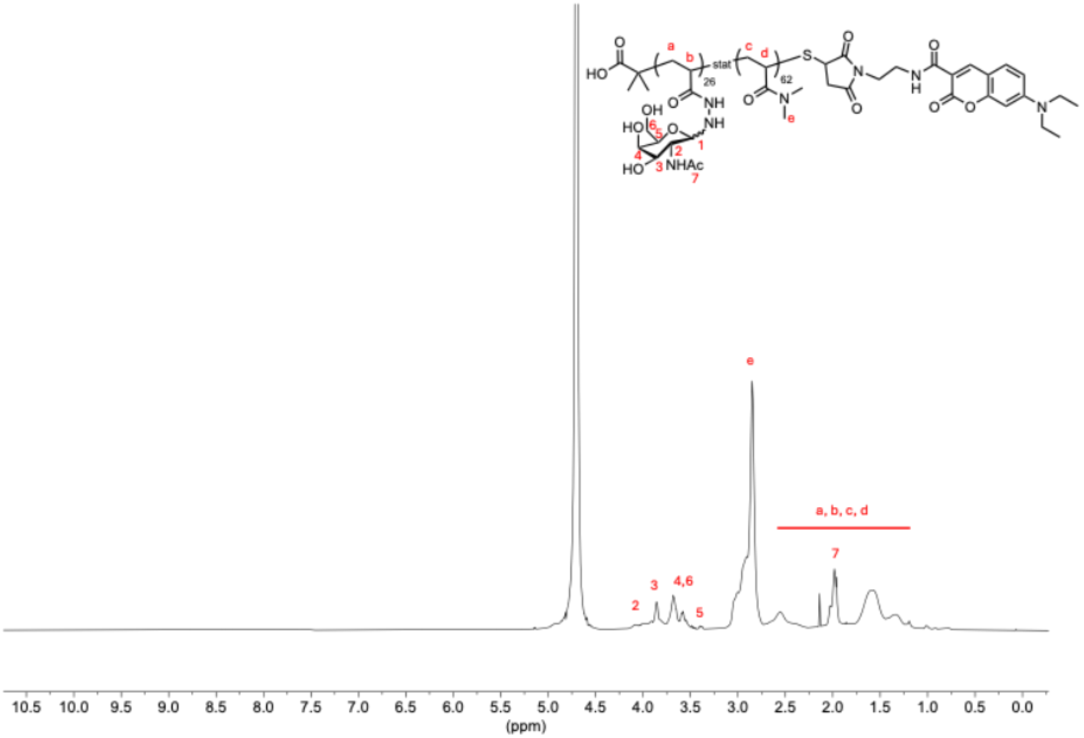

Figure S11: ${}^{1}H$ NMR spectrum (400 MHz, D_2O) of **P3-B**.

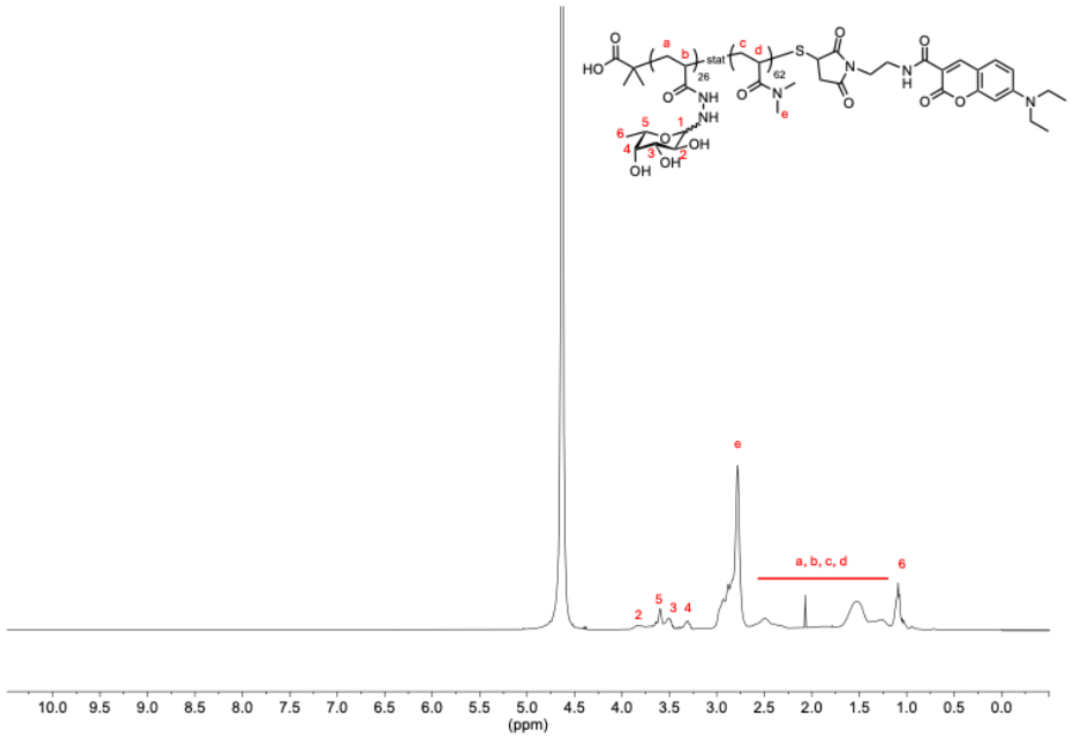

Figure S12: ^{1}H NMR spectrum (400 MHz, D_2O) of **P3-C**.

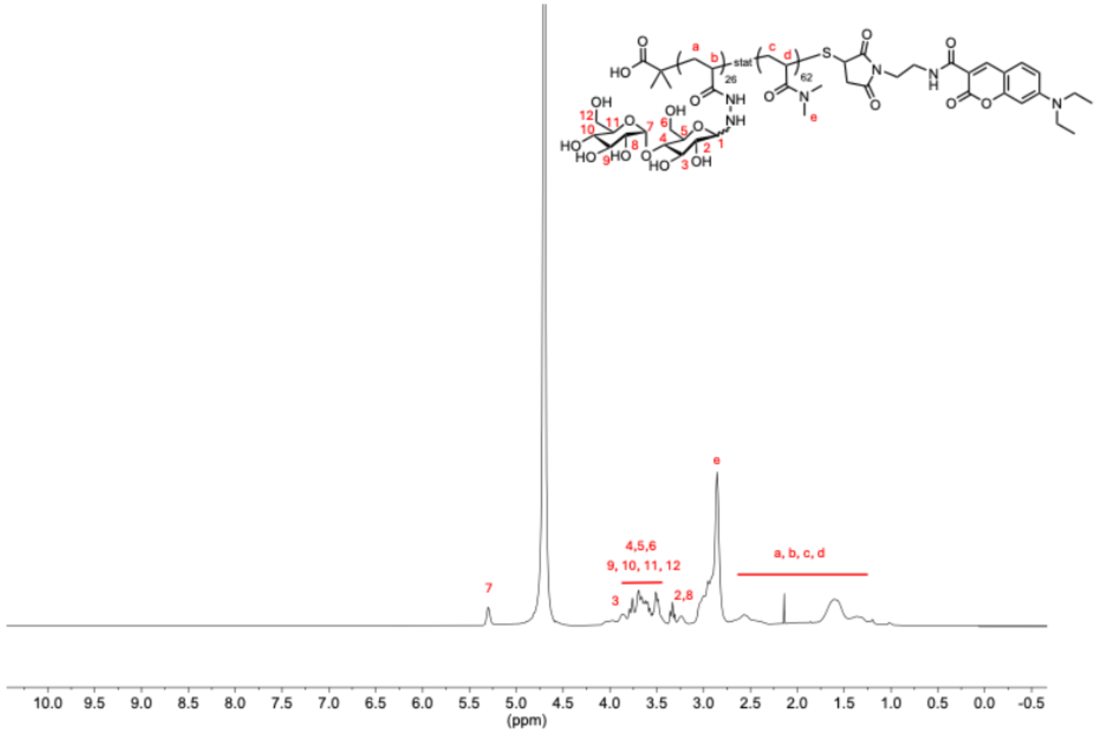

Figure S13: ^{1}H NMR spectrum (400 MHz, D_2O) of **P3-D**.

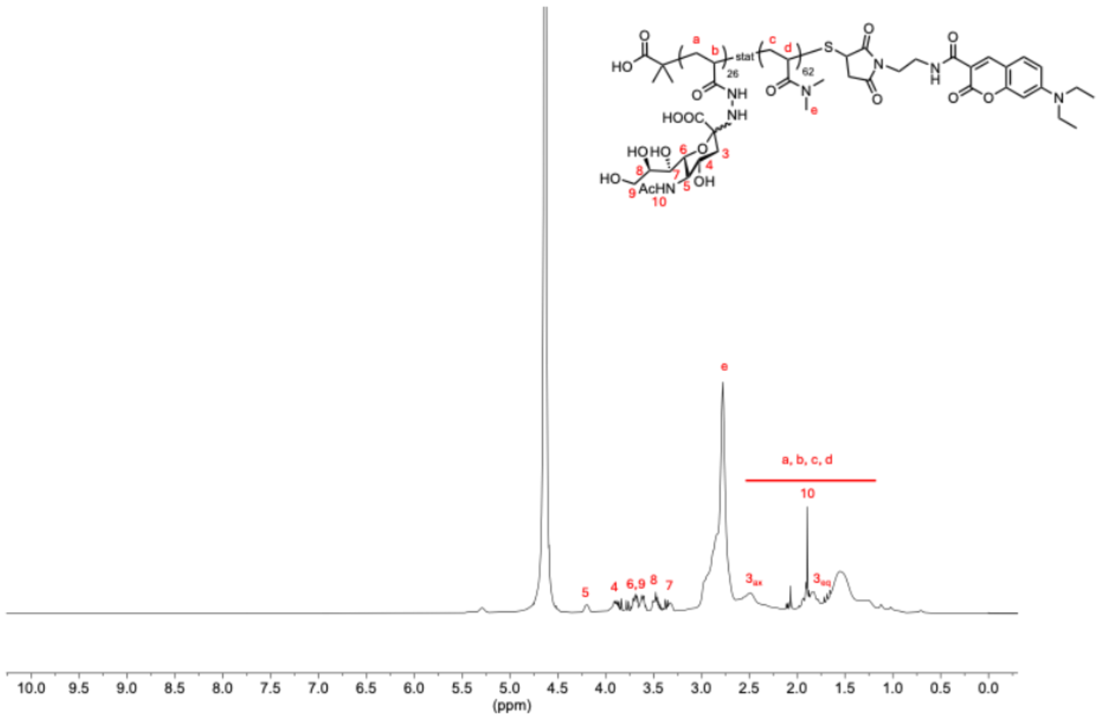

Figure S14: ^{1}H NMR spectrum (400 MHz, D_2O) of **P3-E**.

S2.2 Expression and purification of LTB

Cells from a glycerol stock of *Vibrio sp60* harbouring plasmid pMMB68 [11] (kindly provided by Prof. Tim Hirst) were used to inoculate growth medium (100 mL, 25 g/L LB mix, 15 g/L NaCl, ampicillin 100 μg/mL). The culture was grown overnight at 30 °C with shaking at 200 rpm, then used to inoculate fresh growth medium (6 × 1 L, 25 g/L LB mix, 15 g/L NaCl, ampicillin 100 μg/mL). These cultures were incubated at 30 °C with shaking at 200 rpm until A_{600} reached 0.6 before protein expression was induced by addition of isopropyl β-D-1-thiogalactopyranoside to a concentration of 0.5 mM. Cultures were incubated (30 °C, 200 rpm) for a further 24 h, then cells were removed by centrifugation (7500 *g*, 15 min). The combined supernatant was treated with ammonium sulphate (550 g/L) and left to stir at 5 °C for 2 h. Crude protein was isolated by centrifugation (17,000 *g*, 25 min) and redissolved in 100 mM NaH_2PO_4, pH 7.0, 500 mM NaCl (60 mL). Insoluble material was removed by centrifugation (5000 *g*, 10 min) before the solution was passed through a 0.22 μm filter then loaded onto a lactose-sepharose 6B column and eluted with 300 mM lactose, 100 mM NaH_2PO_4, pH 7.0, 500 mM NaCl. LTB was dialysed against PBS (137 mM NaCl, 2.7 mM KCl, 10 mM Na_2HPO_4, 1,8 mM KH_2PO_4), pH 7.4, lyophilised and stored at -20 °C.

S2.3 Discrimination of Model Lectin Library

Method

Surface chemistry and experimental protocols are provided in the Methods section of the manuscript. Eight replicates for each glycopolymer were chosen randomly from the dataset and used in analysis below. The raw shift data was analysed according to Equation S1:

$$\Delta shift = mean\ after\ lectin\ addition - \ mean\ before\ lectin\ addition \qquad \text{(Eqn. S1)}$$

Where the mean before and after lectin addition was calculated over 20 data points to reduce the impact of noise. The data obtained from this analysis is tabulated in Table S5 and shown graphically in Figure S15.

Data

Table S5: Resonance shift changes (in pixels) of polymer-coated photonic array cells upon lectin addition.

	P3-A	P3-B	P3-C	P3-D	P3-E
**ConA**	-6.6337429	-3.7290619	-3.7290619	-17.04211	-6.0338952
	-5.5610143	-2.6187333	-2.6187333	-27.80699	-5.9848143
	-5.1975286	-1.5705714	-1.5705714	-23.433881	-4.5557524
	-7.3144429	-4.2847762	-4.2847762	-24.186095	-2.8236619
	-6.0208714	-6.5843429	-6.5843429	-23.39001	-3.9825952
	-5.1841952	-3.9835762	-3.9835762	-31.284748	-3.6490238
	-6.7556381	-4.3119524	-4.3119524	-33.058857	-3.086519
	-5.5171952	-4.0729143	-4.0729143	-27.899081	-3.3027429
**WGA**	-34.591886	-19.16141	-2.4703048	-0.6256381	-0.3923762
	-38.27861	-14.918948	-4.0341381	-1.2090286	-0.200181
	-29.965995	-18.738929	-2.1713048	-1.4549095	-2.5938
	-28.690233	-27.117167	-3.0435095	-0.5555095	-1.2164095
	-40.038733	-21.33121	-3.4809333	-1.6424381	-3.4507
	-38.813076	-26.986057	-3.0829333	-1.921081	-1.2914238
	-44.485462	-29.163324	-1.8218571	-0.7651095	-2.1835048
	-45.438962	-26.61251	-1.6406095	-0.5458	6.53797619
**LTB**	-1.9542762	-2.7734905	-4.1792333	-2.8826667	-1.7990952
	-3.0364286	-3.0784333	-3.8282762	-2.0980619	-3.289919
	-3.4417	-3.548581	-4.5170952	-3.3785238	-0.6535333
	-3.0644333	-3.6480143	-4.4427048	-4.8212524	-3.1821857
	-3.7026048	-3.5216286	-4.7444333	-3.9477762	-1.9015952
	-2.6843429	-3.055781	-5.0381381	-5.4850143	-2.0834
	-5.1933857	7.9022619	-6.1955429	-3.8446286	-2.4120143
	-4.1060286	-4.1593048	-6.0105095	-3.2393619	-1.8309333
**SBA**	-0.8969905	-3.0262905	-2.1419667	-0.5268905	-0.4975381

	-0.990619	-2.2980714	-0.9628524	-0.171319	0.00931429
	-1.0833381	-2.6096095	-0.6635905	-0.8167619	-0.0463952
	-1.2722048	-2.5073095	-1.0644333	-1.0388857	-4.1198
	-1.0697571	-3.2629476	0.01048571	-0.6662429	-1.4269714
	-0.9791238	-2.7913143	-0.0466429	-0.6651714	-0.8208619
	-0.3503238	-3.997081	-0.861081	-3.1991571	-0.8213429
	-0.5896381	-0.3496238	-2.3771905	-0.5766286	-0.6601381
**PNA**	-0.3328762	-0.5063476	-0.483419	-0.5339286	2.37919524
	-0.6535619	-0.2380714	1.3814381	-0.2743238	0.41937143
	-0.4289095	-0.0162714	-1.2795238	-0.346981	1.94449524
	-0.0937143	-0.306	-0.1758857	-1.1874143	-0.2424381
	-0.3233857	0.3409	-0.6870714	-0.6841048	0.07874286
	-0.7212143	-0.375319	-0.1266762	-0.9510238	-0.1457571
	-0.864819	-0.0608429	0.32213333	-4.6472143	-0.1573857
	-1.251919	-1.1578857	-0.2009095	-1.1455429	-0.2041762

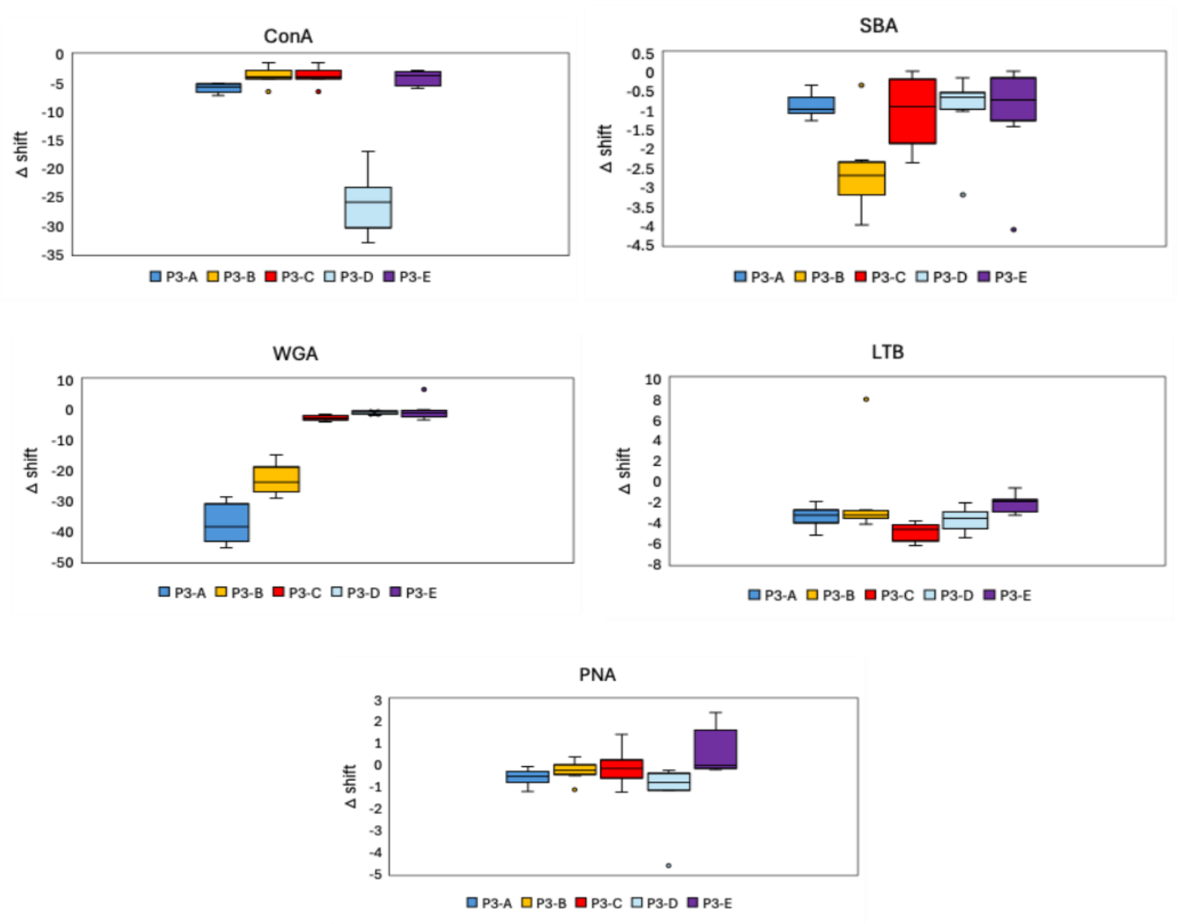

Figure S15: Box and whisker plots of the shift changes tabulated in Table S5 for the polymers **P3-A** to **P3-E** upon lectin addition.

Statistical Analysis

Principal component analysis (PCA) and linear discriminant analysis (LDA) were conducted in IBM SPSS Statistics (version 29.0.0.0 (241)). The principal component method was used to determine the suitability for factor analysis. The KMO measure of sampling adequacy was 0.560, and the Bartlett's Test of Sphericity had a significance <0.001. LDA analysis grouped replicates by response to each lectin, with the intensity changes of the 5 glycopolymers as independent variables. Leave-one-out cross validation analysis was performed by deriving discriminant functions from all other cases and using those to assign the remaining datapoint to a class. Hold-out validation was conducted on the dataset using 5 datapoints for each strain to construct an LDA scoring model which was used to classify the remaining 3 datapoints for each strain (Table S5; green). The hold-out dataset was classified with 100% accuracy (Table S8).

Table S6: Canonical discriminant function coefficients for shift data obtained through LDA analysis.

	**Function**			
**Variable**	1	2	3	4
**P3-A**	0.329	0.012	-0.05	-0.131
**P3-B**	0.127	0.004	0.02	0.227
**P3-C**	0.193	0.234	0.955	-0.131
**P3-D**	0	0.387	-0.118	-0.037
**P3-E**	0.218	0.177	0.096	0.37
**Constant**	4.816	3.549	1.43	0.149

Unstandardised coefficients

Table S7: Assignment matrix detailing results of cross validation leave-one-out analysis for each lectin replicate within the array.

		**Predicted Group**				
		**LTB**	**WGA**	**PNA**	**SBA**	**ConA**
**Actual Group**	**LTB**	8	0	0	0	0
	**WGA**	0	8	0	0	0
	**PNA**	0	0	8	0	0
	**SBA**	0	0	0	8	0
	**ConA**	0	0	0	0	8

Table S8: Assignment matrix detailing results of hold-out validation test set for cGMR array data.

		**Predicted Group**				
		**LTB**	**WGA**	**PNA**	**SBA**	**ConA**
**Actual Group**	**LTB**	3	0	0	0	0
	**WGA**	0	3	0	0	0
	**PNA**	0	0	3	0	0
	**SBA**	0	0	0	3	0
	**ConA**	0	0	0	0	3

Fluorescence array comparison

Method

Solutions of receptors **P3-A**, **P3-B**, **P3-C**, **P3-D**, and **P3-E** were prepared at 5.0 µM concentrations in PBS buffer (pH 7.4) for detection of WGA, SBA, LTB and PNA or 10 mM HEPES, 1 mM $MnCl_2$, 1 mM $CaCl_2$ (pH 7.4) for detection of ConA. Solutions were transferred to a 96-well plate (100 µL/well, 8 replicates per receptor) and emission spectra were recorded (lex 385 nm, lem 410-600 nm, step size 5 nm) (e.g. Fig. S16). A 10 µL aliquot of lectin analyte (150 µM LTB and ConA, 75 µM SBA, PNA, WGA subunit, in appropriate buffer) was added to each well with aspirating and re-dispensing using the micropipette 5 times. Emission spectra were acquired as before. The change in emission in response to lectin addition was calculated using Equation 2:

$$\Delta I = \frac{\int_{600\,nm}^{410\,nm} emission\ after\,lectin\ addition}{\int_{600\,nm}^{410\,nm} emission\ before\ lectin\ addition} \qquad \text{(Eqn. S2)}$$

Outliers were removed from each set of polymer replicates by excluding datapoints >2 SDs from the mean (14 datapoints removed from 200, leaving 7 full replicates of data for each lectin). The data obtained from this analysis is tabulated in Table S9 and shown graphically in Figure S20.

Data

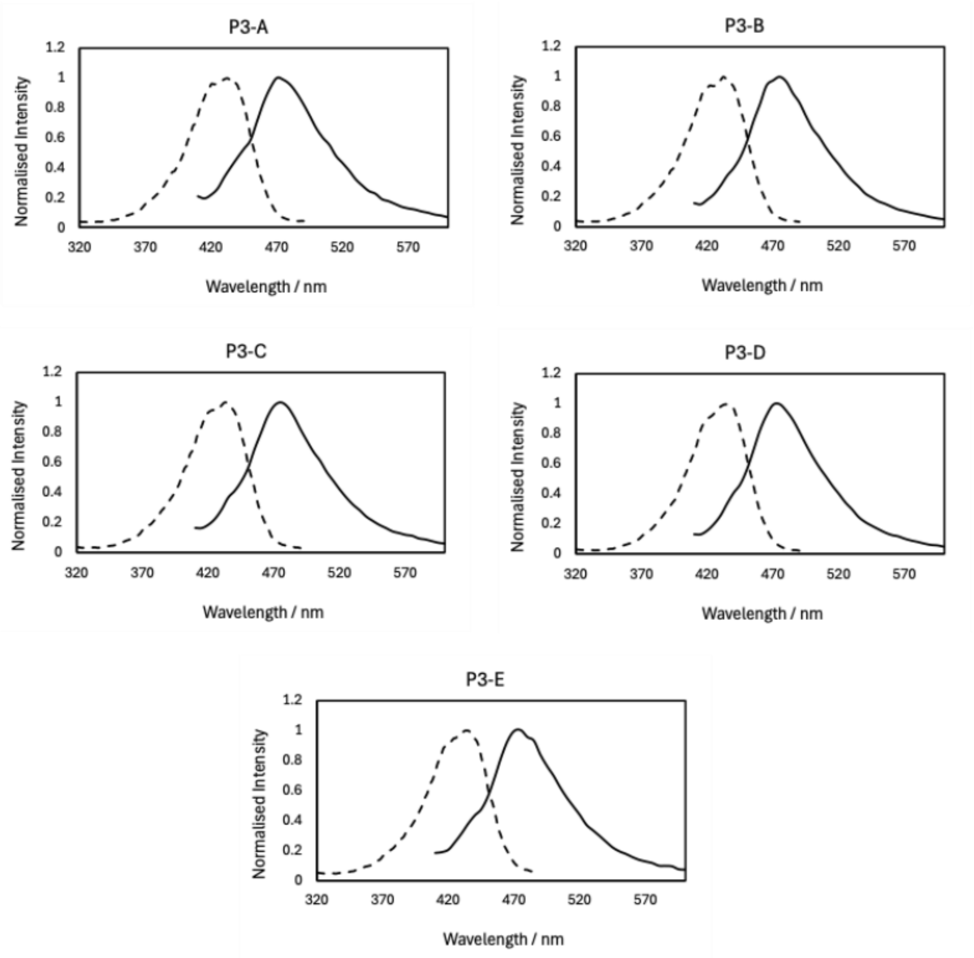

Figure S16: Excitation and emission spectra of polymers **P3-A** to **P3-E** in HEPES buffer (pH 7.4).

Table S9: Emission intensity changes of polymers upon lectin addition as described in Eqn. S2.

	P3-A	P3-B	P3-C	P3-D	P3-E
LTB	1.24096135	1.22356452	1.23184474	1.20686181	1.32300325
	1.21142496	1.228778	1.22406095	1.15854971	1.33466667
	1.21670471	1.20070684	1.22083186	1.13133205	1.3053389
	1.15391609	1.17955416	1.23467	1.15318465	1.30683859
	1.24735343	1.20844001	1.21828872	1.15586168	1.32110156
	1.16589815	1.19019658	1.24671509	1.14518053	1.32271085
	1.19760967	1.17858019	1.21878949	1.16117591	1.31222707
WGA	1.69237598	1.24782347	1.2112729	1.05686785	1.30250154
	1.4423601	1.19122779	1.27123214	1.02189805	1.28976492
	1.43850691	1.25637804	1.30217787	1.085367	1.2881687
	1.52539841	1.2672307	1.1460383	1.05985791	1.32647832
	1.4797689	1.18755204	1.27101876	1.1175816	1.40805267
	1.52685322	1.32072837	1.22405589	1.15125958	1.4079058
	1.52594857	1.42614236	1.14181359	1.08422378	1.38090129
PNA	0.99342948	1.0135446	1.01457558	1.03094652	1.01165385
	0.99809666	1.00279059	0.99398912	1.03325661	1.02389366
	1.03736994	1.01320647	1.00196021	1.050474	1.02395177
	0.98557288	0.99859551	1.11650815	1.01301305	1.02201007
	1.01751592	0.97584585	1.01934836	1.02186157	1.02305507
	1.00459392	1.02055584	1.02251127	1.01801603	1.03728917
	1.00579189	1.0085843	1.0215021	0.98121746	1.02699969
SBA	0.90845186	0.92985006	0.96756744	0.99419437	0.95245883
	0.88788363	0.92972815	0.95642797	0.89688735	0.96994184
	1.01465513	0.93867456	0.93848784	0.86793224	0.9701136
	0.88873399	0.94700338	0.92686511	0.88270374	0.98090297
	0.85231084	0.90725256	0.98164487	0.90329662	0.9603484
	1.00677117	0.9372092	0.924976	0.90834558	1.03489496
	0.92374148	0.90024425	0.9327801	0.90508556	0.94934844
ConA	1.11124031	1.11584306	1.12351735	1.4240173	1.14333345
	1.07974019	1.07342505	1.07269889	1.46441866	1.10049918
	1.07932577	0.99151704	1.04775752	1.44195697	1.12091231
	1.05802542	1.0472918	1.11341188	1.37844812	1.12329065
	1.08741388	1.04076584	1.08269705	1.30080528	1.09923453
	1.0538648	1.02309122	1.08484527	1.36704775	1.09513967

	1.01910338	1.04969188	1.18168477	1.32456496	1.09021845

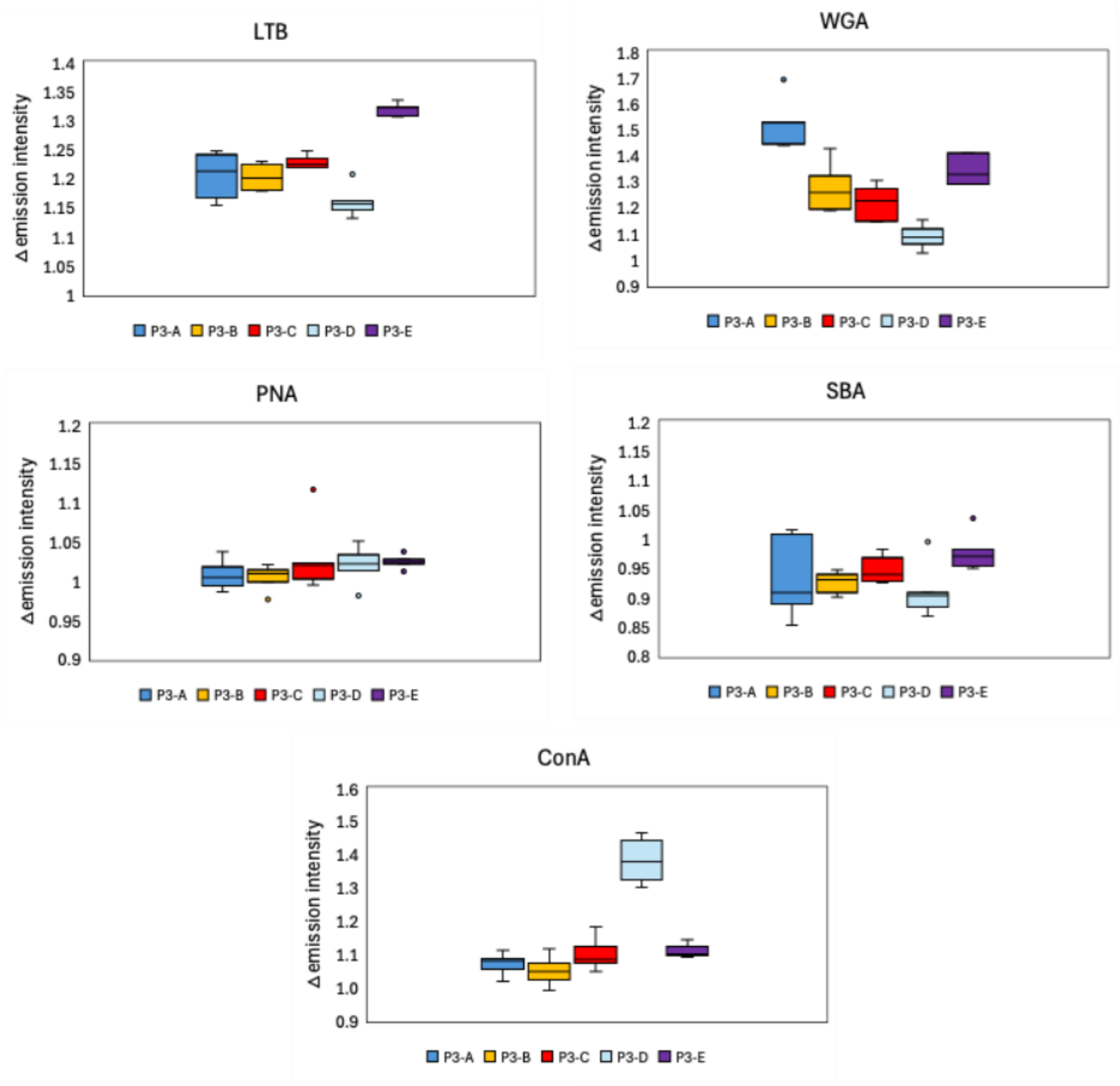

Figure S17: Box and whisker plots of the fluorescence intensity changes tabulated in Table S8 for the polymers **P3-A** to **P3-E** upon lectin addition.

Statistical Analysis

PCA and LDA were conducted in IBM SPSS Statistics (version 29.0.0.0 (241)). The principal component method was used to determine the suitability for factor analysis. The KMO measure of sampling adequacy was 0.818, and the Bartlett's Test of Sphericity had a significance <0.001. LDA analysis grouped replicates by response to each lectin, with the intensity changes of the 5 glycopolymers as independent variables. Leave-one-out cross validation analysis was performed by deriving discriminant functions from all other cases and using those to assign the remaining datapoint to a class. It is interesting to note that although both techniques showed effective discrimination, the fluorescence data produced better separation between classes, whereas the cGMR data showed higher relative magnitude responses for known specific binding pairs, with less noise from non-specific interactions.

Hold-out validation was conducted on the dataset using 4 datapoints for each strain to construct an LDA scoring model which was used to classify the remaining 3 datapoints for each strain (Table S9; green). The hold-out dataset was classified with 100% accuracy (Table S12).

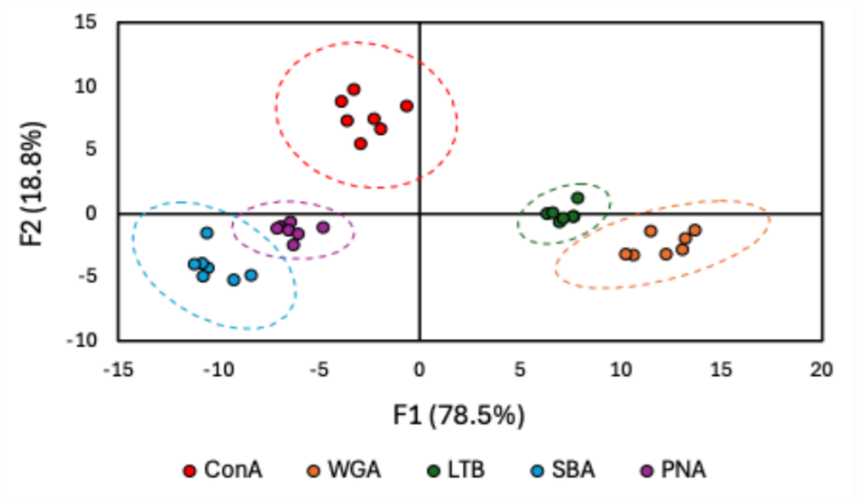

Figure S18: Canonical LDA plot for analysis of the fluorescence response of the 5 **P3** glycopolymers to a selection of lectins (5 µM receptors, pH 7.4). Dashed lines indicate 95% confidence intervals.

Table S10: Canonical discriminant function coefficients for fluorescence data obtained through LDA analysis.

	**Function**			
**Variable**	**1**	**2**	**3**	**4**
**P3-A**	12.359	-0.3	17.072	-3.252
**P3-B**	6.634	-0.118	-0.13	20.158
**P3-C**	20.425	4.41	-0.595	14.111
**P3-D**	-1.846	26.456	3.109	-1.7
**P3-E**	19.957	-10.714	-23.012	-22.325
**Constant**	-64.944	-21.441	4.351	-6.206

Unstandardised coefficients

Table S11: Assignment matrix detailing results of cross validation leave-one-out analysis for each lectin replicate within the array.

		**Predicted Group**				
		**LTB**	**WGA**	**PNA**	**SBA**	**ConA**
**Actual Group**	**LTB**	7	0	0	0	0
	**WGA**	0	7	0	0	0

	**PNA**	0	0	7	0	0
	**SBA**	0	0	0	7	0
	**ConA**	0	0	0	0	7

Table S12: Assignment matrix detailing results of hold-out validation test set for fluorescence data.

		**Predicted Group**				
		**LTB**	**WGA**	**PNA**	**SBA**	**ConA**
**Actual Group**	**LTB**	3	0	0	0	0
	**WGA**	0	3	0	0	0
	**PNA**	0	0	3	0	0
	**SBA**	0	0	0	3	0
	**ConA**	0	0	0	0	3

S2.4 QCMD data confirming surface chemistries

S2.4.1 QCMD: Instrumentation and Materials

QCM-D spectra were recorded on a QSense Pro (Biolin Scientific) using silicon dioxide coated sensors (QSX 303). QCM-D sensors were cleaned by UV–ozone treatment (60 min, 100% power, 5 sccm O_2), followed by sonication in a 2% Hellmanex III solution (Hellma Analytics, Müllheim, Germany) (10 min) and then sonication in ultrapure water (10 min). Cleaned sensors were dried with N_2 then functionalised with polydopamine (15 min, 2 mg/mL dopamine in 5 mM Tris buffer pH 8.5) and loaded into the flow module. The temperature in the QCM-D chamber was set to 21 °C (s.d. < 0.02°C/h) and flow rate was maintained at 20 μL/min for all measurements.

Materials: All chemicals and solvents were used as supplied unless otherwise detailed. Bovine serum albumin (BSA), Casein, dopamine HCl, Tris buffer, PBS, ConA, and WGA were all purchased from Sigma Aldrich. SuperBlock™ was purchased from Thermofisher.

S2.4.2 QCMD: Glycopolymer blocker optimisation

Initial experiments were carried out to identify a suitable blocking strategy to prevent nonspecific binding to the glycopolymer-functionalised sensors. BSA, casein, and Superblock™ were evaluated for preventing nonspecific binding to the control polymer (Table S13), while still allowing lectin binding to the glycopolymers (Table S14). Superblock™ was found to be the optimal blocker, minimising the nonspecific binding of the two exemplar lectins (WGA and ConA) to the control polymer (Table S13, entries 3 and 6).

For the blocker experiments, sensors were prepared with polydopamine as described above, then polymers were attached to the sensor surface via flow inside the QCMD instrument (1 mg/mL polymer, 400 μL per sensor). After a 20 min wash with buffer, the sensor was then blocked using 400 μL per sensor of the relevant blocking solution (BSA at 1 mg/mL, casein at 1% w/v, or Superblock at 10%v/v). After a 40 min wash with buffer, the sensor was then challenged with a solution of the lectin at 50 μg/mL (400 μL per sensor) then a final wash step was carried out with 40 min buffer. Each experiment was carried out in

duplicate. For ConA binding studies 10 mM pH 7.4 HEPES buffer with 1mM $MnCl_2$ and 1mM $CaCl_2$ was used, for WGA studies PBS buffer was used. Surface concentrations of lectins were calculated using the frequency shift from the 7th harmonic with the Sauerbrey equation.[1] We acknowledge that this method of analysis assumes a thin, rigid, and uniform layer, and is therefore only an estimated value.

Table S13: Blocker optimisation for minimising fouling on control polymer (P2). For the purpose of evaluating the blockers, an antifouling scoring criterion was set based on the amount of lectin that fouled the surface: Good < 5 ng/cm^2, moderate 5-10 ng/cm^2, bad > 10 ng/cm^2

Entry	Blocker	Lectin	Mass of lectin bound	Score
1	BSA	WGA	7.4 ng/cm^2, 6.3 ng/cm^2	Moderate
2	Casein	WGA	0.1 ng/cm^2, 0 ng/cm^2	Good
3	Superblock	WGA	1.0 ng/cm^2, 3.3 ng/cm^2	Good
4	BSA	ConA	20.2 ng/cm^2, 17.7 ng/cm^2	Bad
5	Casein	ConA	34.1 ng/cm^2, 37.4 ng/cm^2	Bad
6	Superblock	ConA	2.0 ng/cm^2, 3.0 ng/cm^2	Good

WGA binding to P3-A was successful, with comparable lectin binding densities observed with each of the three blockers (BSA, casein, Superblock™) (Table S14). However, with ConA the binding density was increased on the casein-blocked P3-D surface (compared to BSA- and Superblock-blocked surfaces), this is likely due to the additional fouling of ConA binding non-specifically to the casein as well as the selective binding of the lectin to the glycopolymer.

Table S14: Blocker optimisation: tests with P3-A and P3-D to confirm lectin binding has not been prevented for WGA or ConA

Entry	Polymer	Blocker	Lectin	Mass of lectin bound
1	P3-A	BSA	WGA	53.9 ng/cm^2, 49.9 ng/cm^2
2	P3-A	Casein	WGA	56.9 ng/cm^2, 58.2 ng/cm^2
3	P3-A	Superblock	WGA	46.8 ng/cm^2, 56.4 ng/cm^2
4	P3-D	BSA	ConA	54.1 ng/cm^2, 59.2 ng/cm^2
5	P3-D	Casein	ConA	103.4 ng/cm^2, 83.9 ng/cm^2
6	P3-D	Superblock	ConA	53.6 ng/cm^2, 61.7 ng/cm^2

S2.4.3 QCMD: Glycopolymer surface characterisation

The glycopolymer-functionalised surfaces were characterised using QCMD to: (i) confirm successful polymer immobilisation and (ii) determine the surface concentration of the polymer. For these experiments sensors were prepared with polydopamine as described above, then polymers were attached to the sensor surface via flow inside the QCMD instrument (1 mg/mL polymer, 400 µL per sensor). Experiments were carried out in duplicate, details of the glycopolymer binding densities are provided in Table S15. Surface concentrations of glycopolymers were calculated using the Sauerbrey equation [1]. We acknowledge that this method of analysis assumes a thin, rigid, and uniform layer, and is therefore only an estimated value.

Table S15: Glycopolymer surface densities

Entry	Polymer	Molecular weight (kDa)	Binding mass (surface density)
1	P2	11.4	44.6 ng/cm^2 ($2.4×10^{12}$ $polymers/cm^2$),<br>44.8 ng/cm^2 ($2.4×10^{12}$ $polymers/cm^2$)
2	P3-A	12.3	32.6 ng/cm^2 ($1.6×10^{12}$ $polymers/cm^2$),<br>26.8 ng/cm^2 ($1.3×10^{12}$ $polymers/cm^2$)
3	P3-B	12.3	16.4 ng/cm^2, ($0.8×10^{12}$ $polymers/cm^2$),<br>29.3 ng/cm^2, ($1.4×10^{12}$ $polymers/cm^2$)
4	P3-C	10.8	32.4 ng/cm^2, ($1.8×10^{12}$ $polymers/cm^2$),<br>39.7 ng/cm^2, ($2.2×10^{12}$ $polymers/cm^2$)
5	P3-D	15.9	37.4 ng/cm^2 ($1.4×10^{12}$ $polymers/cm^2$),<br>31.6 ng/cm^2 ($1.2×10^{12}$ $polymers/cm^2$)
6	P3-E	14.6	18.7 ng/cm^2, ($0.8×10^{12}$ $polymers/cm^2$),<br>29.1 ng/cm^2, ($1.2×10^{12}$ $polymers/cm^2$)

S3. Flow cell details

For cGMR measurements the sensors were positioned inside a flow cell (Fig. S19), with a chamber volume of 250 μL. Flow was provided by a syringe pump (Legato® 180 Syringe Pump, Kd Scientific, 788180) via Tygon® tubing (E-3603).

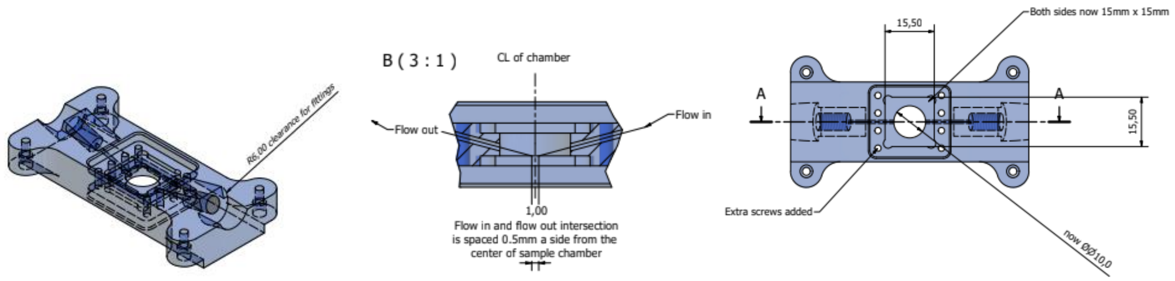

Figure S19: Details of the flow cell used in cGMR measurements

S4. Spotting Optimisation

Spotting of binders onto the sensor array surface was peformed using a SCIENION sciFLEXARRAYER-S3. By programming a map of the array dimensions, each sensor pair can be addressed and spotted individually (Fig. S20a). Spotting outcomes were directly affected by the concentration of the spotting solution. Higher concentrations of binders (≥500 μg/mL in PBS) resulted in film formation on the piezoelectric dispense capillary (PDC), skewing the spotting and thus reducing the spatial accuracy (Fig. S20b). 250 μg/mL was found to be the optimal concentration for the binders used in

this manuscript, as shown in Figure S20(c) this produced uniform droplets and spatially-accurate spotting without film formation on the PDC.

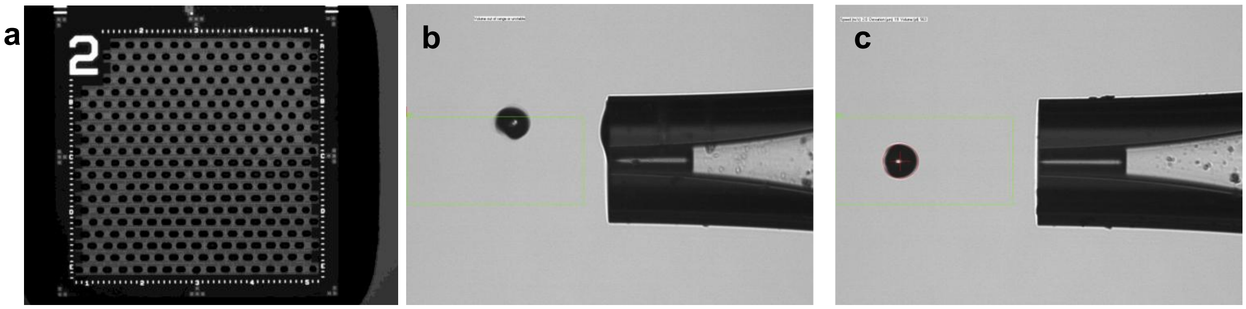

Figure S20. Spotting optimisation (a) spotting map to address each pair of sensors; (b) failed spotting due to film formation; (c) successful spotting.

S5. Different Binders on the Photonic Array

Surface Chemistry used: Briefly, sensors were first coated with PDA, after which biorecognition elements were immobilised by spotting: antibodies and glycopolymers were directly conjugated to PDA followed by blocking (SuperBlock™), whereas biotin-tagged aptamers were attached via a neutravidin layer.

S5.1. Antibody-functionalised Array

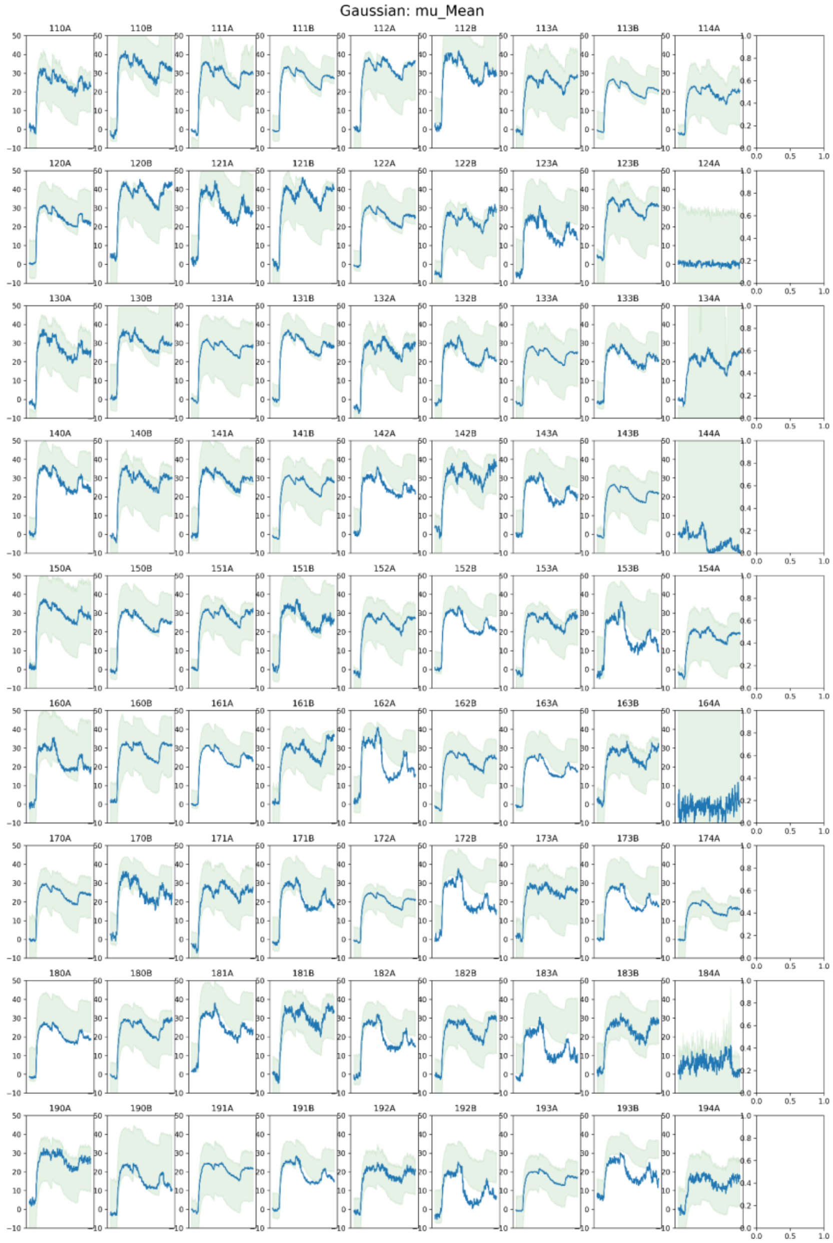

Figure S21: Anti CRP all in flow

The antibody (Fig. S22) functionalised sensors showed a shift in resonance as a solution of 50 μg/mL C-reactive protein (CRP) flowed over the sensor. The test sensors were spotted with antibody, the controls were omitted from spotting and functionalised with blocker only.

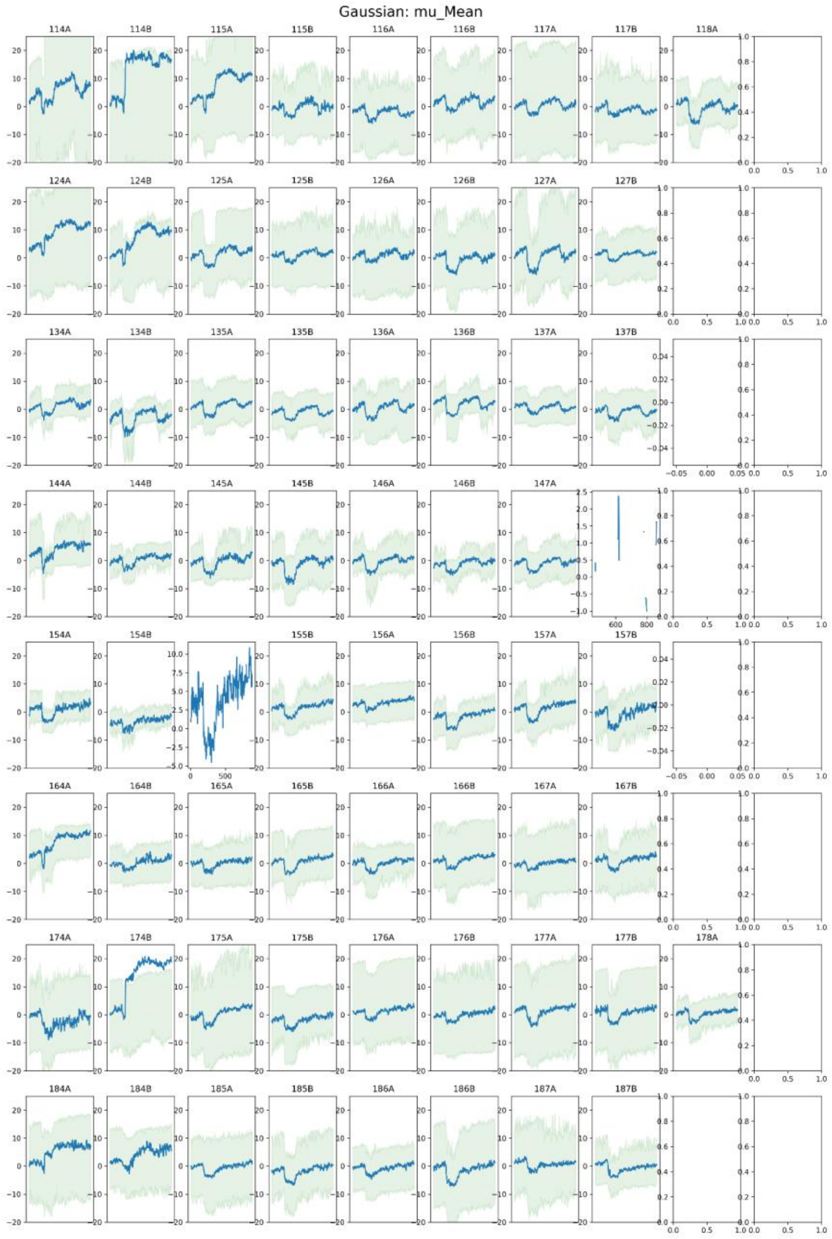

Figure S22: Anti CRP spotted (rows 1-4) vs no antibody (rows 5-8)

S5.2. Aptamer-functionalised Array

CRP aptamer[12]: 5'biotin, GGC AGG AAG ACA AAC ACG ATG GGG GGG TAT GAT TTG ATG TGG TTG TTG CAT GAT CGT GGT TGT GGT GCT GT. The aptamer functionalised sensors showed a shift in resonance as a solution of 50 µg/mL CRP flowed over the sensor (Fig. S23). The test sensors were spotted stepwise with Neutravidin (250 µg/mL) and then aptamer (1 µM), the controls were functionalised with blocker only.

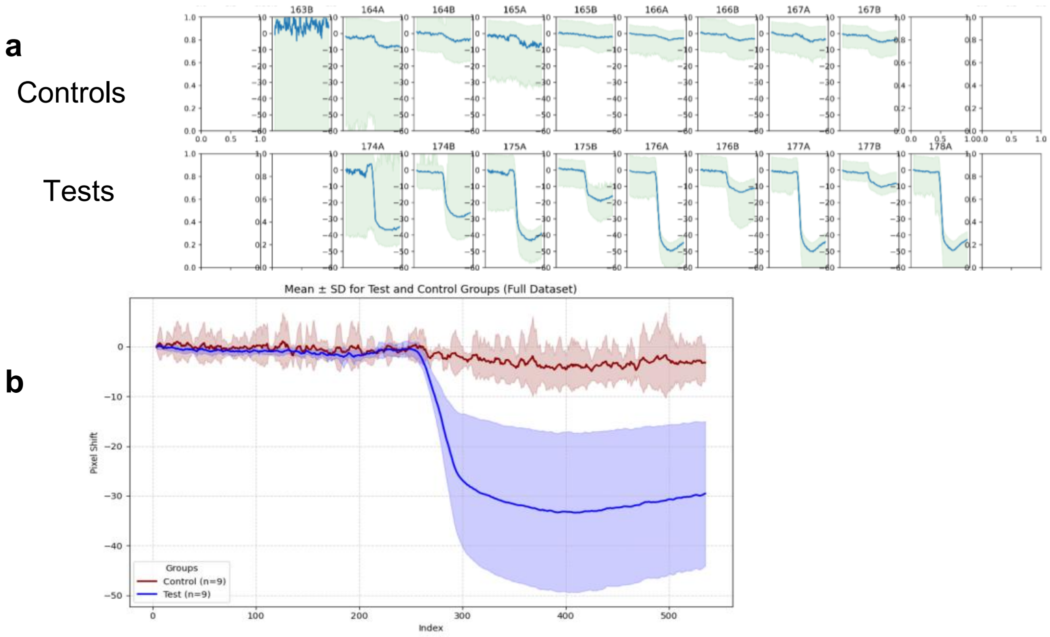

Figure S23: CRP-aptamer: (a) spotted (row 2) vs no aptamer control (row 1); (b) mean plots.

S5.3 Glycopolymers

S5.3.1 WGA Binding to P3-A Dose Curve

Array chips were prepared with P3-A (test), P2 (control) and blocker only (control) sensors via spotting. Once functionalised, sensors were loaded into the flow cell and then the test solution was flowed over while measuring the cGMR response on *Optical Setup 1*. The solutions were prepared with concentrations of WGA in PBS as detailed in Table S16 and the cGMR shifts were plotted in Figure S24.

Table S16: WGA Binding to P3-A

WGA concentration (M)	ΔRIU mean (± s.d.)
2.60E-06	4.77E-03 (±7.40E-04)
1.30E-06	5.60E-03 (±3.46E-04)
6.50E-07	3.96E-03 (±9.05E-04)
2.60E-07	2.97E-03 (±6.33E-04)
1.30E-07	7.43E-04 (±2.75E-04)
2.60E-08	3.23E-04 (±1.29E-04)
2.60E-09	1.25E-05 (±1.08E-04)

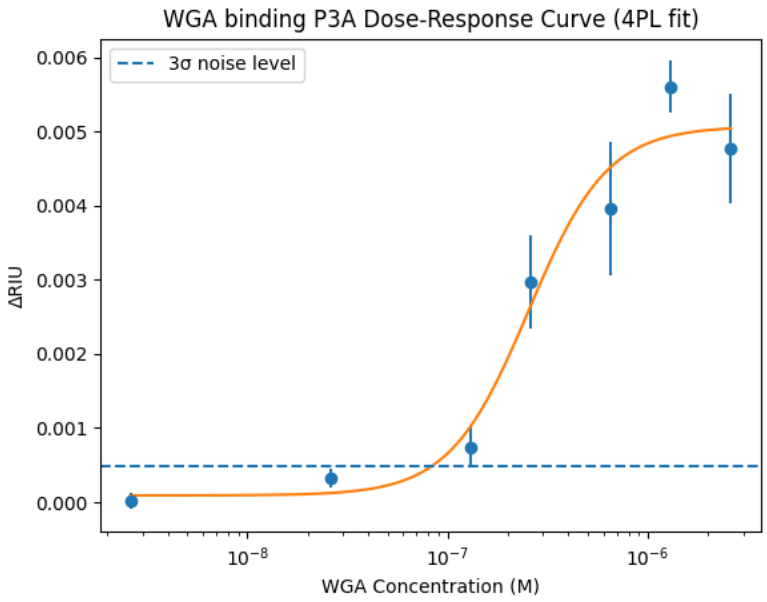

Figure S24: Dose-response analysis of WGA binding to P3-A compared to the P2 control.

S5.3.2 Glycopolymers in Human Plasma

Array chips were prepared with P3-A (test), P2 (control) and blocker only (control) sensors via spotting. Once functionalised, sensors were loaded into the flow cell and then the test solution was flowed over while measuring the cGMR response on *Optical Setup 1*. The solutions were prepared with 50 ug/mL WGA in either PBS or x10 diluted human plasma (Sigma Aldrich P9523, x10 diluted with PBS).

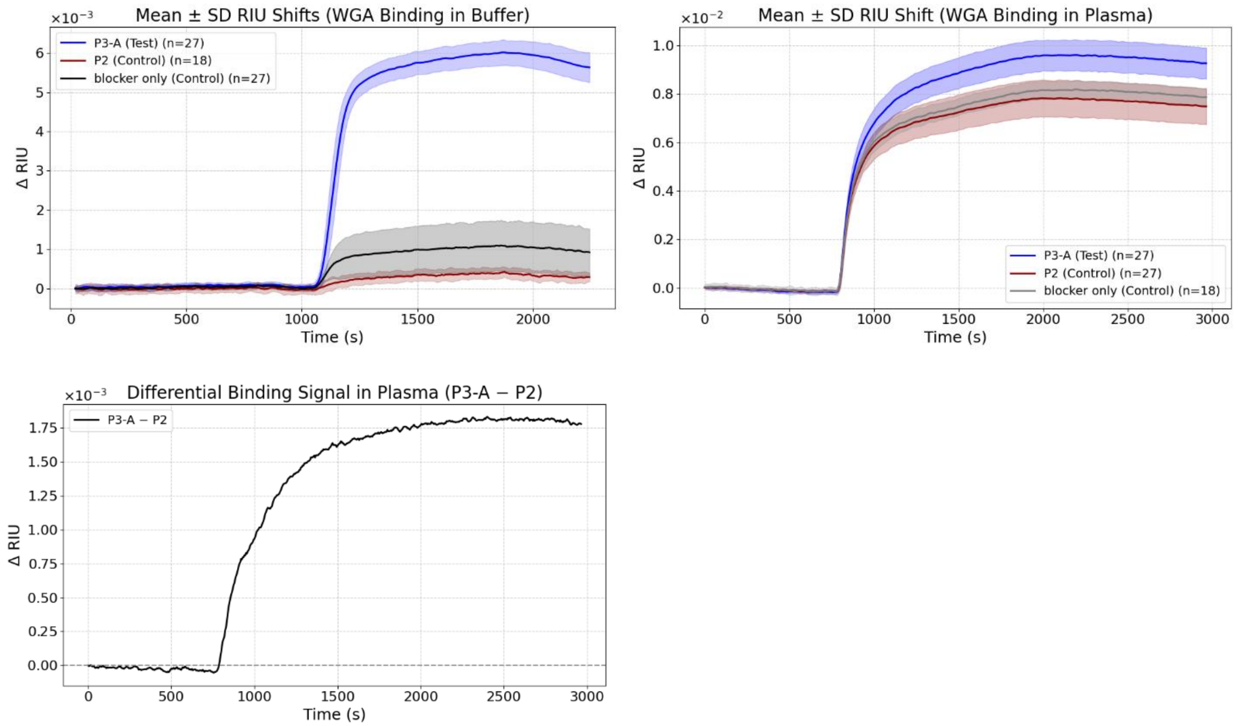

Figure S25: Glycopolymer-lectin binding interactions in complex matrix: (a) binding of WGA in buffer; (b) binding of WGA in human plasma; (c) differential plot of P3-A (test) minus P2 (control) for binding in human plasma.

S6. Comparison of the cGMR array with Established Platforms

Table S17: Comparison of SPR imaging and Genalyte with this cGMR array.

Metric	Typical SPR Imaging (CCD-based)	Genalyte Maverick (Ring Resonator Platform)	cGMR Array
**Form Factor**	Large Benchtop	Compact Benchtop	Handheld/portable (*Optical setup 3*)
**Weight**	~50-100 kg	~14 kg	<1 kg
**Environment**	Controlled Lab (Vibration sensitive)	Clinic	Field/Point of care
**Instrument cost**	$150-$450k+	$50-$150k	<$1k
**Cost per Test**	$200-$500/chip	$50-$100/chip	<$5/chip
**Field of View (FOV)**	5-10 × 5-10 mm (25–100 mm^2)	Not imaging-based. Chip is 1 mm × 6 mm	5 × 5 mm (25 mm^2) on (*Optical Setup 2*)
**ROI Count**	100-500 standard<br>Up to 2000 is possible	128 standard<br>256 high-density version	322 pairs, 644 discrete sensors
**ROI Size**	50-200 μm (diameter)<br>0.0025-0.04 mm^2	~10–30 μm ring resonators	250 × 100 μm<br>0.025 mm^2
**Frame Rate**	1-10 fps	N/A (continuous readout, ~1–10 Hz equivalent)	2.93 fps
**Sensitivity**	~10^{-6} to 10^{-5} RIU	~10^{-6}-10^{-4} RIU equivalent	~10^{-4} RIU
**Dynamic Range**	0.05-0.1 RIU	0.01-0.05 RIU	0.06 RIU
**Optical Mode**	Plasmonic<br>(metal-dielectric)	Photonic<br>(silicon ring resonators)	Photonic<br>(grating resonance)

S7. References

1. G. Sauerbrey, “The Use of Quartz Oscillators for Weighing Thin Layers and for Microweighing,” Zeitschrift für Physik, Vol. 155, No. 2, 1959, pp. 206-222
2. Lee et al., “Polydopamine Surface Chemistry: A Decade of Discovery” ACS Appl. Mater. Interfaces, 2017
3. Jiří Homola, Surface Plasmon Resonance Sensors for Detection of Chemical and Biological Species *Chemical Reviews* **2008** *108* (2), 462-493 DOI: 10.1021/cr068107d
4. Muhammad A. Butt Surface Plasmon Resonance-Based Biodetection Systems: Principles, Progress and Applications—A Comprehensive Review. Biosensors 2025, 15(1), 35; https://doi.org/10.3390/bios15010035
5. Z. Liu, J. Hu, J. Sun, G. He, Y, Li, G, Zhang, *J. Polym. Sci. A.,* 2010, 48, 3573-3586.
6. M. Xu, J. Qian, A. Suo, W. Xu, R. Liu and H. Wang, *RSC Advances*, 2015, 5, 20890-20899.
7. X. Kuang, G. Liu, X. Dong, X. Liu, J. Xu, D. Wang, *J. Polym. Sci. A.,* 2015, 53, 2095-2103.
8. T. L. Foley, A. Yasgar, C. Garcia, A. Jadhav, A. Simeonov, M. D. Burkart. *Org. Biomol. Chem.,* 2010, 8 ,4601-4606.
9. A. Kaur, M. A. Haghighatbin, C. F. Hogan, E. J. New, *Chem. Commun.,* 2015, 51, 10510-10513.
10. J. E. T. Corrie, *J. Chem. Soc., Perkin Trans. 1,* 1994, 2975-2982.
11. Leece, R.; Hirst, T. R., Microbiology, 1992, 138 (4), 719-724.
12. Huang, C. J. *et al.* Biosens. Bioelectron. 2010, 25 (7), 1761-1766.
